# Breaking the vicious cycle of diabetic wounds with an exosome-engineered dual-responsive microneedle patch

**DOI:** 10.1016/j.mtbio.2026.103198

**Published:** 2026-05-08

**Authors:** Xinyu Gu, Shen Shen, Qingmiao Shi, Ziyi Xu, Minghang Zhang, Lifan Zhang, Chen Xue, Yuting He, Juan Lu, Li Li

**Affiliations:** a Henan Key Laboratory of Cancer Epigenetics, Cancer Institute, The First Affiliated Hospital, College of Clinical Medicine, Medical College of Henan University of Science and Technology, Luoyang, 471003, China; b Department of Infectious Diseases, The First Affiliated Hospital of Zhengzhou University, Zhengzhou University, Zhengzhou, 410052, China; c Department of Infectious Diseases, The First Affiliated Hospital, College of Clinical Medicine, Medical College of Henan University of Science and Technology, Luoyang, 471003, China; d State Key Laboratory for Diagnosis and Treatment of Infectious Diseases, National Clinical Research Center for Infectious Diseases, China-Singapore Belt and Road Joint Laboratory on Infection Research and Drug Development, National Medical Center for Infectious Diseases, Collaborative Innovation Center for Diagnosis and Treatment of Infectious Diseases, The First Affiliated Hospital, Zhejiang University School of Medicine, Hangzhou, 310003, China; e Department of Emergency, Henan Provincial People's Hospital, People's Hospital of Zhengzhou University, Zhengzhou, 450003, China

**Keywords:** Diabetic wounds, Microneedles, Exosomes, Anti-inflammation

## Abstract

Diabetic wound (DW), a prevalent type of chronic non-healing injury, poses substantial clinical challenges owing to persistent oxidative stress, dysregulated inflammation and recurrent bacterial infections. To rationally modulate the microenvironment of DWs, this study fabricated a core-shell structured multifunctional microneedle (MN) patch, designated as AEP-GCMN. Specifically, we designed engineered exosomes, Aloe-Exo^PC^ (AEP), by encapsulating proanthocyanidins (PC) into aloe-derived exosomes, which were then integrated into methacrylated hyaluronic acid (HAMA) to serve as the core layer of the MN patch. In contrast, a polyvinyl alcohol/polyvinylpyrrolidone (PVA/PVP) blend was loaded with catalase (CAT) and surface-functionalized with Gold Nano-Stars (GNS), forming the structural shell of the patch. The AEP-GCMN patch operates through a multi-stage mechanism: its casing rapidly produces oxygen via CAT upon wound contact, while the embedded GNS enable photothermal antibacterial therapy under NIR light. Subsequently, the sustained release of AEP leads to the intracellular delivery of PCs, which alleviate oxidative stress and inhibit inflammation to improve the microenvironment. Additionally, Aloe-Exos contribute to angiogenesis and cell migration. This intelligent responsive system offers a synergistic strategy for the temporal modulation of hypoxia, infection and chronic inflammation in DWs, representing a promising intelligent therapeutic approach for DW management.

## Highlights

• A core-shell microneedle patch is engineered for intelligent microenvironment-responsive therapy of diabetic wounds.
• The catalytic shell rapidly decomposes H_2_O_2_ to generate oxygen, alleviating wound hypoxia upon application.
• The bilayer design achieves spatiotemporally regulated sequential release of multiple therapeutic agents.
• Engineered plant-derived exosomes actively suppress inflammation, oxidative stress and ferroptosis.
• Gold nanostars mediate potent near-infrared photothermal antibacterial activity and immunomodulation.

## Graphical abstract

**ToC figure** Proposed multi-mechanistic actions of AEP-GCMN in DW therapy: anti-inflammatory, pro-angiogenic, antibacterial, and overall wound-healing promotion.

**Figure:**
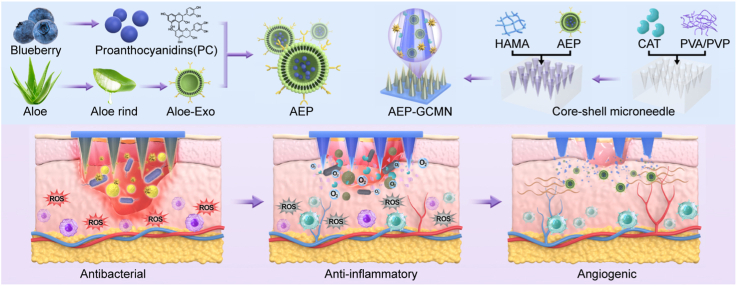

## 1 Introduction

DWs, which are chronic non-healing wounds resulting from diabetes, are characterized by their persistent failure to heal, leading to high rates of amputation and mortality while imposing a substantial economic and healthcare burden on medical systems [1]. Normal wound healing is an orderly and predictable biological process that follows established stages of hemostasis, inflammation, proliferation, and remodeling, ultimately achieving complete tissue repair [2]. Unfortunately, the distinctive hyperglycemic environment in DWs exacerbates oxidative stress and dysregulates the inflammatory response [3]. This pathological microenvironment further leads to impaired vascularization, microbial infection, and wound bed hypoxia, ultimately impeding the transition of wound repair from the inflammatory phase to the proliferation and remodeling phases, thereby delaying wound healing [4]. In recent years, local delivery systems based on bioactive materials have emerged as a research frontier in the treatment of DWs, and MN patches, which can encapsulate a wide range of factors and bioactive substances, represent a promising and effective therapeutic strategy in this field [5]. MN patches enable painless and precisely targeted drug delivery through transdermal penetration of their needle tips, and then realize multifunctional therapeutic approaches by loading different components [6]. However, the rational design of MN structures and the construction of MN systems for loading novel bioactive components to achieve multi-dimensional regulation of the DW microenvironment remain a research area of high academic value.

Proanthocyanidins (PC), a class of polyphenolic compounds derived from natural plants, demonstrate substantial therapeutic potential owing to their exceptional antioxidant activity and pleiotropic biological functions [7]. This class of polyphenolic compounds, polymerized from flavan-3-ol units, is ubiquitously distributed in grape seeds, pine bark, and certain berries [8]. Studies have demonstrated that procyanidin B2 (PCB2), the core component of PC, enhances the antioxidant capacity of endothelial cells by activating the transcriptional function of nuclear factor erythroid 2-related factor 2 (Nrf2) [9], while reduction of oxidative stress can directly downregulate the excessive activation of pro-inflammatory signaling pathways, such as NF-κB, and modulate macrophage polarization, ultimately alleviating chronic inflammation [10]. Furthermore, Chen et al. demonstrated that PCs can modulate the expression of glutathione peroxidase 4 (GPX4), a ferroptosis-related protein, thereby suppressing ferroptosis [11]. Nevertheless, the chemical instability of PCs under physiological conditions leads to their rapid degradation [12], and the low in vivo bioavailability of these compounds necessitates rationally engineered nanocarriers to enable their precise targeting and sustained release. Exosomes (Exo), as cell-derived nanovesicles, have emerged as highly promising carriers in the field of drug delivery owing to their inherent biocompatibility, low immunogenicity, capacity to traverse biological barriers, and cell homing properties [13].

In recent years, cell-derived Exos have emerged as pivotal mediators of intercellular communication, providing a promising cell-free therapeutic strategy. These nanoscale extracellular vesicles encapsulate a diverse array of proteins, lipids, and nucleic acids (miRNAs, mRNAs), which can directly modulate the biological functions of recipient cells, including migration, proliferation and differentiation [14]. Furthermore, in comparison to conventional animal-derived Exos, plant-derived Exos exhibit distinct advantages including broad source availability, low immunogenicity, and scalability for large-scale production [15]. Increasing numbers of plant-derived Exos—including lemon-derived Exos [16], ginseng-derived Exos [17], and potato-derived Exos [18]—have exhibited notable therapeutic potential in the management of refractory diseases. *Aloe vera*, a traditional medicinal plant, has been extensively documented to possess potential wound-healing efficacy via its pulp-derived gel, which exerts a positive regulatory effect on cell migration and proliferation [19]. Current research indicates that the biological activity of Aloe is not only attributed to its known chemical components but is more likely closely related to the natural Exos secreted by aloe cells [20]. Building upon this premise, this study prepared a novel engineered Exo, designated as Aloe-Exo^PC^ (AEP), via the functional integration of PCs—a class of compounds with well-characterized antioxidant and anti-inflammatory activities—with aloe-derived Exos.

At present, most Exo-based disease treatment systems are designed as single structures or loaded with multiple therapeutic agents. Although these systems exhibit certain therapeutic effects, they often lack the spatiotemporal programmability required to address the dynamic and multifaceted characteristics of the disease microenvironment. To overcome this limitation and achieve multidimensional therapy for DWs, it is essential to design a hydrogel MN delivery system that combines intelligent responsiveness with structural heterogeneity. In recent years, Zhang et al. developed a photothermal-responsive porous hollow MN, which exhibits superior antibacterial activity and wound-healing promoting properties [21], and Wang et al. developed a pH-responsive composite multifunctional MN patch for the management of myocardial infarction [22]. Luan et al. developed a bionic meta microneedle photonic crystal patch that enables continuous, noninvasive monitoring of wound infection-related metabolites while delivering therapeutic agents [23]. One of the persistently present endogenous pathological signals in DWs is excessive reactive oxygen species (ROS) dominated by hydrogen peroxide (H_2_O_2_), which can be specifically recognized and catalyzed by CAT. Upon contact with the enzyme's active center in the wound microenvironment, CAT binds specifically to H_2_O_2_ molecules, leading to the generation of water and oxygen [24], and this catalytic specificity for the characteristic hyperoxidative stress in DWs represents a pivotal determinant for realizing intelligent MNs. Additionally, for differential distinction of multi-drug loading in MN patches, the construction of core-shell structured MNs with spatial partitioning capabilities constitutes a practical fabrication strategy, which enables precise modulation of DW healing. Studies have demonstrated that the core-shell structural design of MNs facilitates the sequential controlled release and functional synergy of multiple active components [25] [26]. This structurally differential design enables the MN patch to exert precise intervention on the complex wound microenvironment.

In summary, such a system should ideally: (i) immediately respond to endogenous wound signals to alleviate early hypoxia and combat infection, and (ii) subsequently enable sustained, on-demand delivery of therapeutic Exos to modulate the late-stage inflammatory and redox microenvironment. To this end, we herein propose a core–shell structured MN patch with a dual-responsive, multi-component design. The shell layer, composed of a rapidly dissolving PVA/PVP matrix loaded with CAT and surface-functionalized with GNS, is engineered to provide an immediate response upon wound contact: CAT enzymatically generates oxygen to relieve hypoxia, while GNS enable on-demand photothermal antibacterial therapy under NIR. Following shell dissolution, the core layer, comprising a HAMA hydrogel encapsulating engineered Exos (AEP), serves as a sustained-release depot. This core layer ensures the prolonged, intracellular delivery of PCs and aloe-derived exosomal cargoes to mitigate oxidative stress, reprogram macrophage polarization, and promote angiogenesis. This spatiotemporally programmed, core–shell synergistic strategy is designed to sequentially break the vicious cycle of DWs, representing a significant advance over conventional monolithic systems. We believe that this sequential synergistic therapeutic strategy with dual intelligent responsiveness provides a multi-dimensional and intelligent microenvironmental modulation solution for DW management.

## 2 Results and discussion

### 2.1 Synthesis and characterization of the AEP-GCMN microneedle system

**Fig. 1:**
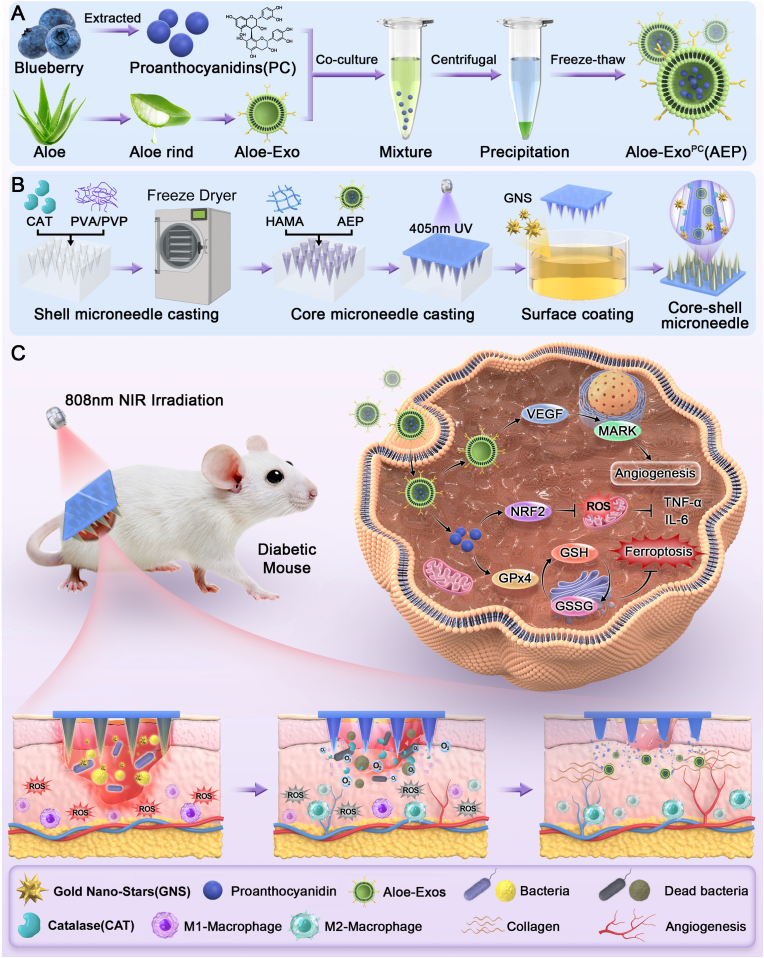
Schematic illustration of the synthesis and proposed mechanism of the AEP-GCMN patch. (A) Preparation of engineered exosomes (Aloe-Exo^PC^) from *Aloe vera* and proanthocyanidins (PCs). (B) Fabrication process of the core–shell structured AEP-GCMN patch. (C) Proposed multi-mechanistic actions of AEP-GCMN in DW therapy: anti-inflammatory, pro-angiogenic, antibacterial, and overall wound-healing promotion.

**Fig. 2:**
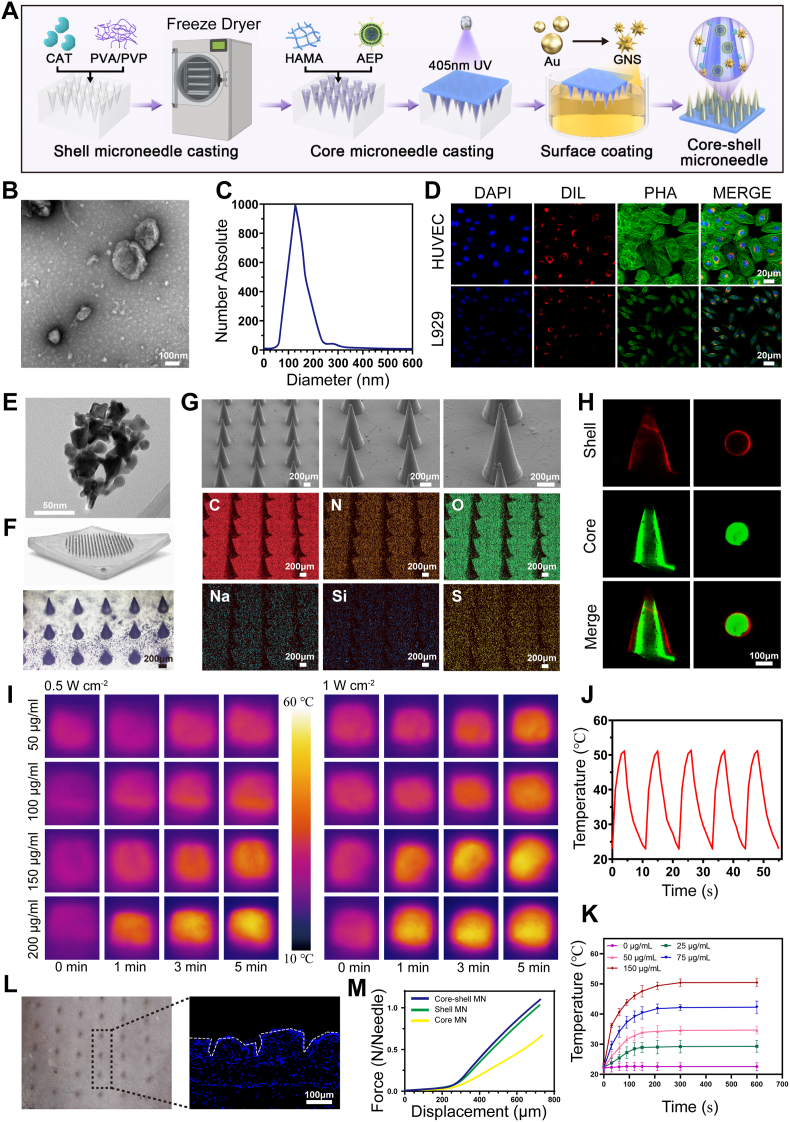
Synthesis and characterization of the AEP-GCMN core–shell microneedle system. (A) Schematic illustration of AEP-GCMN fabrication. (B) TEM image of engineered exosomes (Aloe-Exo^PC^, AEP). (C) Size characterization of Aloe-Exo by Nanoparticle Tracking Analysis. (D) Confocal image showing cellular uptake of Dil-labeled AEP (red) by HUVECs. Nuclei: blue (DAPI); cytoskeleton: green (phalloidin). (E) TEM image of synthesized gold nanostars (GNS). (F) Macroscopic view of the microneedle patch. (G) SEM image of a single microneedle and corresponding elemental mapping. (H) Fluorescence image of the core–shell structure (core: red; shell: green). (I) Infrared thermal images of MNs loaded with different GNS concentrations under NIR irradiation. (J) Photothermal heating curves over five on-off cycles. (K) Temperature-time profiles under NIR irradiation. (L) Optical image of MN penetration into mouse skin. (M) Mechanical force-displacement curve of the core–shell MNs. ∗*P* < 0.05, ∗∗*P* < 0.01 and ∗∗∗*P* < 0.001. (For interpretation of the references to color in this figure legend, the reader is referred to the Web version of this article.)

This study successfully designed and fabricated an intelligent responsive MN patch with a core-shell structure, termed AEP-GCMN, aiming to treat diabetic infected wounds through a spatiotemporal synergy strategy (Fig. 1). As illustrated in Fig. 2A, the system integrated multiple functional units: the core layer consisted of HAMA hydrogel loaded with engineered exosomes, AEP, designed for the sustained release of therapeutic exosomes; the shell layer consisted of a PVA/PVP composite matrix, encapsulating CAT, and GNS that were loaded onto the shell via the immersion method. This layer was responsible for the rapid response to the wound microenvironment and endowed the system with NIR photothermal conversion capability.

First, we successfully prepared and characterized the core active component, the engineered exosomes AEP. Natural exosomes (Aloe-Exos) were isolated from aloe vera gel via differential centrifugation. Leveraging their membrane permeability, PCs were efficiently loaded into these exosomes via an incubation method to form AEP. Transmission electron microscopy (TEM) observation showed that the resulting AEP exhibited the classic saucer or cup-shaped vesicle structure (Fig. 2B). Nanoparticle Tracking Analysis (NTA) characterization confirmed the nanosized nature of Aloe-Exo, showing a mean particle size of 157 nm and a peak concentration at 132 nm (Fig. 2C). The loading efficiency of PC into Aloe-Exo was quantitatively evaluated. As summarized in Supplementary Table 1, the drug loading content (DLC) and encapsulation efficiency (EE) were determined to be 14.66 ± 0.69% and 19.82 ± 1.44% (mean ± SD, n = 3), respectively. These results confirm the successful and reproducible loading of PC into the exosomal carriers via the incubation method. Fig. 2D demonstrated the superior cellular internalization capacity of AEP. Confocal microscopy imaging results revealed that following co-incubation of Dil-fluorescently labeled AEP with HUVECs, the red fluorescence signal, representing AEP successfully internalized into the cytoplasm, was colocalized with the cytoskeleton (green) and cell nuclei (blue), thus confirming the efficient cellular uptake of AEP. As shown in Fig. 2E, GNS synthesized via the seed-mediated growth method exhibited a uniform multi-branched star-like morphology with multiple sharp tips under TEM. This structural feature facilitated enhanced NIR light absorption and improved photothermal conversion efficiency [27].

The macroscopic photograph of the AEP-GCMN patch fabricated in this study displayed a well-aligned MN array. (Fig. 2F). As shown in Fig. 2G, SEM characterization results demonstrated that the AEP-GCMN patch exhibited a well-defined geometric morphology with a distinct conical structure—featuring straight needle shafts, sharp tips, and smooth surfaces. Further dimensional measurements revealed that the MN tip height was approximately 720 μm and the base width was around 300 μm, with a rationally designed taper. These geometric parameters ensured that the MN array could effectively penetrate the skin stratum corneum upon application of appropriate pressure, thereby facilitating transdermal drug delivery [28]. Moreover, elemental mass spectrometry analysis further confirmed the uniform distribution of elements mainly composed of C, N, and O in the MN patch, and the Au element was also widely distributed on the surface of the MN, which is closely related to the coating of GNS on the microneedle surface (Fig. S2). To visually validate the successful construction of the core-shell structure, we employed fluorescent dyes of distinct colors—NHS-Cy5 and FITC—to label the shell and core layers of AEP-GCMN (Fig. 2H), respectively. Fluorescence microscopy images clearly demonstrated a typical core-shell architecture, where the red core layer was fully encapsulated by the green shell layer. A well-defined interface was observed between the two layers, with no interpenetration detected, and the colocalization analysis of fluorescence staining further confirmed this result (Fig. S3).

As shown in Fig. 2I, this study systematically evaluated the effect of GNS solutions at varying concentrations—loaded within MN patches—on the patches’ photothermal conversion performance. Under irradiation with an 808 nm NIR laser, GNS-loaded MNs exhibited a significant, concentration-dependent temperature elevation effect. Specifically, the microneedle patch loaded with 200 μg/mL GNS achieved a maximum local temperature of 50.76 °C after 5 min of irradiation (Fig. 2K). This temperature fell within a range previously shown to effectively suppress common wound pathogens, while inducing no observable damage to the normal cells surrounding the wound site [29]. Moreover, the heating curves highly overlapped after five “on-off” cycles, demonstrating excellent photothermal stability (Fig. 2J).

To evaluate the mechanical properties of the AEP-GCMN patch, three types of MNs were fabricated, single shell MN, single core MN and core-shell structured MN. Compression test was conducted to determine the full-needle compressive force of each group. The results indicated that the compressive forces of the single shell MN and core-shell structured microneedles were 1.17 N/needle and 1.23 N/needle, respectively, with no statistically significant difference observed between the two groups. In contrast, the compressive force of MN composed of HAMA was only 0.68 N/needle, which was significantly lower than those of the other two groups (Fig. 2M). This difference could be attributed to the lower biological hardness of HAMA compared to the PVA/PVP composite matrix [30]. These findings suggested that the core-shell structure enhanced mechanical strength to the MN patch, enabling it to penetrate the skin with ease. Concurrently, the mouse skin penetration assay verified that AEP-GCMN could effectively penetrate skin tissue, forming intact microchannels that remained after MN removal. Fluorescence microscopy images clearly visualized the formation of micropores within the dermal layer following MN insertion (Fig. 2L).

To verify that the fabrication process did not compromise the enzymatic function of CAT, and to assess whether the subsequent photothermal treatment affects its activity, we quantitatively compared the H_2_O_2_-decomposing activity of free CAT, CAT released from AEP-GCMN patches, and CAT released from AEP-GCMN patches subjected to NIR irradiation (AEP-GCMN + NIR). Over the 60-s measurement period, the relative H_2_O_2_ concentration rapidly and comparably decreased in the free CAT, AEP-GCMN, and AEP-GCMN + NIR groups, whereas no significant reduction was observed in the blank control (Fig. S1). Statistical analysis of the residual H_2_O_2_ at 60 s confirmed that there was no significant difference in catalytic efficiency among the free CAT, AEP-GCMN, and AEP-GCMN + NIR groups. These results conclusively demonstrate that neither the microneedle fabrication process nor the subsequent NIR-induced photothermal treatment compromises the enzymatic activity of CAT. This is attributable to the intrinsic thermal stability of CAT, which retains its structural integrity and catalytic function upon brief exposure to temperatures below 55–60 °C. Given that the maximum photothermal temperature reached in our system is approximately 50.8 °C, the mild heating does not denature the enzyme or impair its active site. Consequently, the AEP-GCMN shell layer achieves a synergistic dual function without mutual interference: GNS-mediated photothermal antibacterial action operates in parallel with CAT-catalyzed oxygen generation, jointly alleviating infection and hypoxia during the early phase of wound treatment [31].

In addition to mechanical strength and enzymatic activity, the in vitro release kinetics of PC from the core layer were systematically evaluated. The results revealed that the AEP-GCMN system possesses distinct sustained-release characteristics, enabling prolonged drug delivery in a simulated physiological environment. This sustained behavior ensures the continuous action of active ingredients at the wound site and effectively prevents premature drug depletion. The corresponding cumulative release curve is presented in Fig. S4 in the Supporting Information, which further substantiates the rationale of our core-shell structural design for diabetic wound management. The local temperature of the AEP-GCMN reached approximately 50.76 °C under NIR irradiation, yet this mild thermal stimulation did not compromise the integrity of the encapsulated exosomes. Previous studies have reported that exosomes typically maintain structural and drug-loading stability within the 50 to 55 °C range due to their unique natural lipid and protein compositions. Furthermore, the superior biological activities observed in the NIR treatment group during subsequent cellular assays and in vivo wound repair further validate that the photothermal process did not diminish the therapeutic potency of AEP. Moderate photothermal effects have also been demonstrated to optimize exosomal delivery efficiency by improving local microcirculation and enhancing cell membrane permeability, thereby creating a synergistic enhancement with the bioregulatory effects of AEP [32].

### 2.2 In vitro synergistic antibacterial and biofilm disruption evaluation

**Fig. 3:**
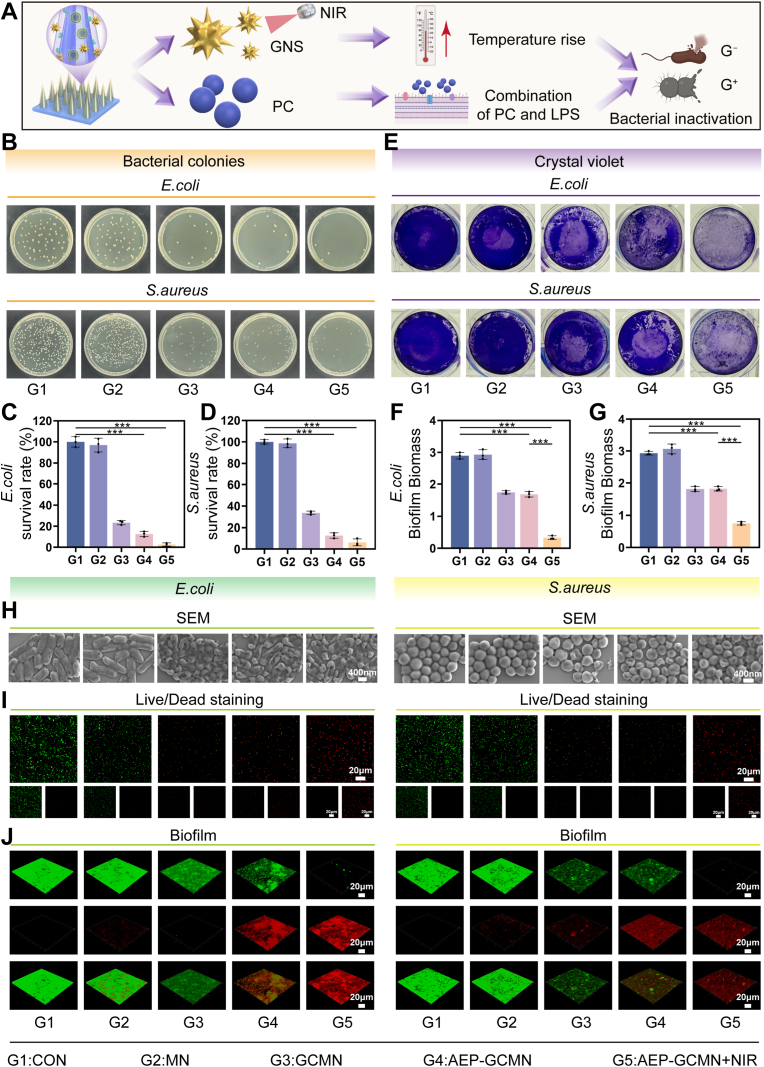
In vitro antibacterial and antibiofilm activity of AEP-GCMN. (A) Schematic of the synergistic bactericidal mechanism combining GNS-mediated photothermal therapy and PC-induced membrane disruption. (B) Photographs of bacterial colony formation on agar plates. (C, D) Quantitative analysis of survival rates for *E. coli* (C) and *S. aureus* (D). (E) Representative images of crystal violet-stained biofilms. (F, G) Quantitative analysis of biofilm biomass for *E. coli* (F) and *S. aureus* (G). (H) SEM images showing morphological damage to bacterial cells. (I) Live/Dead staining of bacteria (green: live; red: dead). (J) Confocal laser scanning microscopy (CLSM) images of biofilms (green: SYTO 9; red: PI). ∗*P* < 0.05, ∗∗*P* < 0.01 and ∗∗∗*P* < 0.001. (For interpretation of the references to color in this figure legend, the reader is referred to the Web version of this article.)

Bacterial infection is a core pathological factor hindering the healing of DWs. To address this challenge, the AEP-GCMN microneedle system constructed in this study aims to achieve efficient antibacterial effects through the spatiotemporal synergy between its shell and core components. As illustrated in Fig. 3A, upon application, the microneedle rapidly releases GNS and CAT from its shell, while the core enables sustained release of PCs. Under NIR light irradiation, GNS generate a localized photothermal effect. Simultaneously, the released PC can bind to components such as lipopolysaccharides (LPS) on bacterial cell membranes [33]. This dual physical and chemical mechanism synergistically disrupts the membrane structure of both Gram-positive (*S. aureus*) and Gram-negative (*E. coli*) bacteria, thereby exerting broad-spectrum bactericidal activity.

To validate this synergistic antibacterial effect, we systematically evaluated five experimental groups (Group 1: blank control; Group 2: blank MN; Group 3: GCMN MN; Group 4: AEP-GCMN MN; Group 5: AEP-GCMN MN + NIR). Fig. 3B visually exhibited abundant colony formation in Groups 1 and 2, a reduction in colonies in Group 3, and very few colonies in Groups 4 and 5. Quantitative statistics further confirmed (Fig. 3C and D) that compared to the control group, Group 4 (AEP-GCMN) significantly reduced the survival rates of both bacterial types (*E. coli:*11.12%, *S. aureus:* 12.35%), indicating that the drug-loaded microneedle itself possessed certain antibacterial activity. However, Group 5 (AEP-GCMN + NIR) demonstrated nearly complete bactericidal efficacy, with bacterial survival rates dropping to extremely low levels (*E. coli:* 0.52%, *S. aureus:* 3.71%), highlighting the crucial synergistic value of the NIR-triggered photothermal effect combined with the chemical action of PCs.

To further investigate its ability to eradicate the more resilient biofilms prevalent in chronic infections, we performed crystal violet staining. Fig. 3E indicated that the biofilm biomass was least in Group 5. Quantitative analysis (Fig. 3F and G) indicated that Group 5 achieved the highest clearance rates for both EC and SA biofilms, significantly outperforming all other groups. Subsequently, SEM was employed to examine the microscopic morphological alterations of *E. coli* and *S. aureus* subjected to different grouping treatments. As illustrated in Fig. 3H, both bacterial strains treated in Group 5 exhibited severe membrane shrinkage, rupture, or even dissolution, with complete structural collapse, and bacteria in Groups 3 and 4 also exhibited a certain degree of structural impairment, whereas control bacteria remained intact with smooth surfaces. Furthermore, the live/dead bacterial staining fluorescence images (Fig. 3I) correlated with these findings, showing dense green fluorescence (live bacteria) in the control group, while the field of view in Group 5 was predominantly red fluorescence (dead bacteria), clearly demonstrating potent bactericidal capacity.

Finally, three-dimensional confocal imaging of biofilms revealed that untreated biofilms were thick, densely structured, and predominantly composed of live bacteria (green). Following combined treatment in Group 5, the biofilms became significantly thinner, sparse, and fragmented, with dead bacterial (red) signals dominating (Fig. 3J). In summary, in vitro antibacterial experiments, spanning multiple levels from colony counts and biofilm quantification to microscopic morphology and three-dimensional structure, demonstrated that the AEP-GCMN system could efficiently eliminate both Gram-positive and Gram-negative bacteria and their biofilms, particularly when activated by NIR light. This is achieved through a synergistic mechanism combining photothermal ablation mediated by GNS and chemical disruption driven by PCs [34] [35]. The photothermal effect rapidly induces localized hyperthermia, leading to bacterial protein denaturation, membrane fluidity disruption, and eventual physical disintegration [36]. Simultaneously, PCs exert multimodal chemical interference, including membrane permeabilization, enzyme inhibition, and suppression of virulence factors. Together, these dual actions not only enhanced immediate bactericidal efficacy but also mitigate the risk of bacterial resistance development. Compared with other recently reported antibacterial strategies for DWs, our AEP-GCMN system exhibits superior efficacy. For instance, Younis et al. developed a green-synthesized silver nanoparticle (AgNP) system using the cyanobacterium Synechocystis sp., which demonstrated antibacterial activity with an inhibition zone comparable to chloramphenicol [37]. Similarly, Chen et al. developed a GelMA/AA/Cu hydrogel dressing with Cu^2+^-mediated antibacterial properties for DW healing [38]. Our system achieves a much higher bacterial killing rate (>96%) and superior biofilm clearance (>95%) through the complementary physical and chemical dual mechanisms, which also mitigate the risk of bacterial resistance—a concern not addressed by conventional metal nanoparticle or metal ion-based approaches.

### 2.3 Cell proliferation, migration, and pro-angiogenic activity of the AEP-GCMN microneedle system

**Fig. 4:**
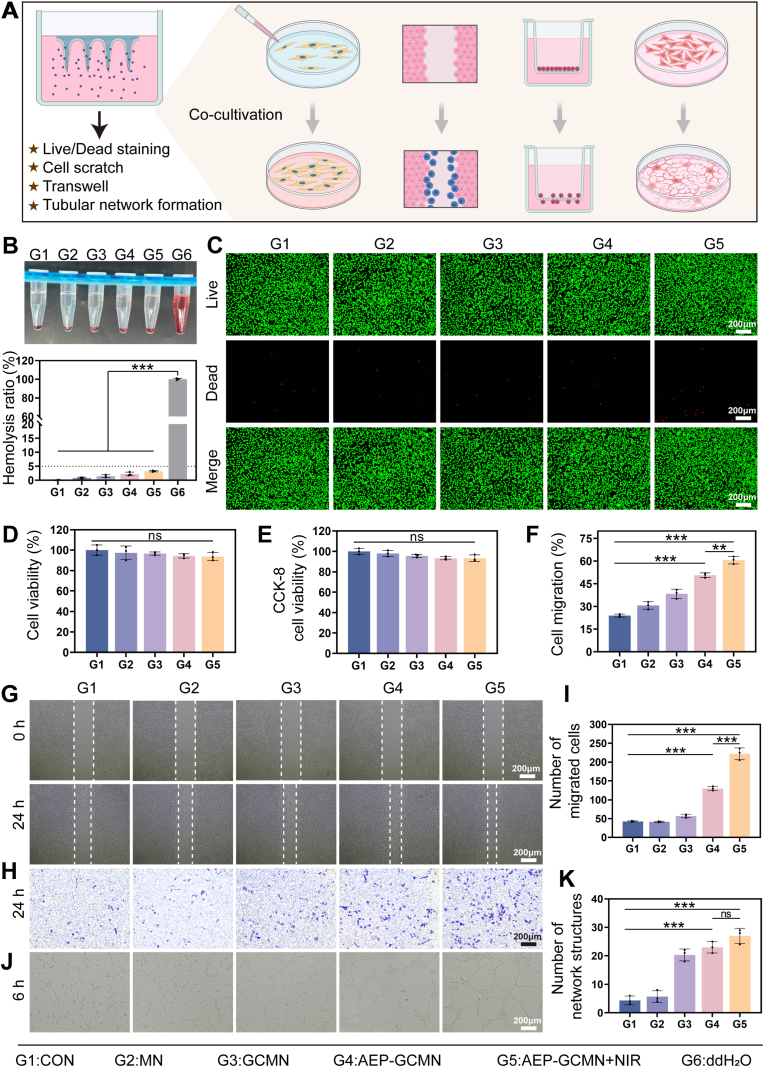
Biocompatibility and pro-angiogenic effects of AEP-GCMN extracts on HUVECs. (A) Schematic of the in vitro experimental setup. (B) Hemolysis assay (G6: ddH_2_O, positive control). (C) Live/Dead staining after 72 h. (D) Quantitative analysis of (C). (E) CCK-8 assay for cell proliferation. (F) Quantitative analysis of cell migration from the scratch assay. (G) Representative images of the scratch assay at 24 h. (H) Representative images of the Transwell assay at 24 h. (I) Quantitative analysis of migrated cells from (H). (J) Representative images of tube formation assay at 6 h. (K) Quantitative analysis of tube formation. ∗*P* < 0.05, ∗∗*P* < 0.01 and ∗∗∗*P* < 0.001.

To systematically evaluate the biocompatibility of the AEP-GCMN patch and its crucial regulatory effects on vascular endothelial cell function, a series of in vitro functional assays were performed by co-culturing human umbilical vein endothelial cells (HUVECs) with extracts from the MNs (Fig. 4A). Firstly, the hemolysis assay demonstrated that the hemolysis rates induced by extracts from all microneedle groups (G1-G5) except the positive control group were below 5%, which meet the international safety standard for biomaterials [39]. These rates showed no statistically significant difference from the negative control group (G6, normal saline), while the positive control group (ddH_2_O) caused complete hemolysis (Fig. 4B). This preliminarily confirmed the excellent blood compatibility of the MN system. To assess the cytocompatibility and proliferative activity of the MN patches. Live/dead cell staining and corresponding quantitative analysis demonstrated that after 72 h of culture, cells in all experimental groups (G2-G5) maintained high viability (green fluorescence) with no evident cytotoxicity compared to the blank control group (Fig. 4C and D). The results of the CCK-8 assay further demonstrated that the cell survival rates of groups G1 to G5 were 97.32%, 96.01%, 95.37%, 94.16%, and 94.08%, respectively. These findings indicated that the composition of the AEP-GCMN system exerted a negligible effect on cell proliferation. (Fig. 4E).

Cell migration is a critical step in angiogenesis and wound re-epithelialization. As shown in Fig. 4G, the G5 group exhibited the highest cell migration capacity, which was significantly superior to that of the other groups. Quantitative calculation of cell migration rates across groups revealed the following results: the migration rates of G1, G2, and G3 were 23.68%, 32.97%, and 39.18%, respectively, with no statistically significant differences observed among these groups. In contrast, the cell migration rate of the G4 group (50.29%) was significantly enhanced; notably, the G5 group treated with NIR irradiation (61.10%) displayed even better performance than the G4 group (Fig. 4F). Moreover, the results of the Transwell migration assay showed that the G4 and G5 groups exhibited the highest number of cells migrating through the matrix, indicating that AEP-GCMN effectively promotes the directional migration ability of endothelial cells. It is noteworthy that the pro-migratory effect was most pronounced in the G5 group (Fig. 4H and I), suggesting that the mild photothermal effect mediated by NIR may act as a positive synergistic factor with the biological activity of AEP, potentially by transiently enhancing cell membrane fluidity or activating migration-related signaling pathways [40].

The pro-angiogenic potential was assessed using an endothelial cell tube formation assay [41]. As depicted in Fig. 4J, following 6 h of incubation, only incomplete tubular networks were observed in Groups G1, G2, and G3. In contrast, Groups G4 and G5 exhibited enhanced tube formation capacity, characterized by the formation of denser, more highly branched, and structurally intact capillary-like networks—with the most prominent improvement noted in Group G5. Additionally, the total number of network structures was found to be significantly increased across these groups (Fig. 4K). This provided robust evidence that the engineered Exos, with AEP continuously released from their core layer, are the core functional units driving angiogenesis—where these aloe-derived functional delivery vesicles play a pivotal role [42]. The localized mild photothermal effect exhibits a dual regulatory mechanism driven by the differential thermal sensitivities of bacteria and mammalian tissue cells. The elevated temperature directly compromises bacterial membrane integrity and significantly amplifies the bactericidal efficacy of the released therapeutic agents. Concurrently this mild hyperthermia provides a favorable biophysical stimulation to the surrounding healthy tissue by enhancing local blood perfusion and upregulating the expression of proangiogenic factors [43]. This distinct cellular response ultimately enables the designed intelligent patch to eradicate persistent infections while simultaneously accelerating the reconstruction of the vascular network.

In summary, this series of in vitro cell experiments systematically demonstrates the excellent biocompatibility and bioactivity of the AEP-GCMN microneedle system. Its functional core lies in the sustained release of AEP from the core layer, which efficiently mimics key steps of physiological angiogenesis by promoting endothelial cell proliferation, migration, and tube formation. The photothermal effect from the shell layer (under NIR) may act as a physical enhancer, further optimizing the cellular microenvironmental response. This combined strategy provides a solid theoretical and experimental foundation for repairing the impaired vascular network in DWs at the in vivo level [44].

### 2.4 In vitro anti-inflammatory and macrophage reprogramming activity of the AEP-GCMN microneedle system

**Fig. 5:**
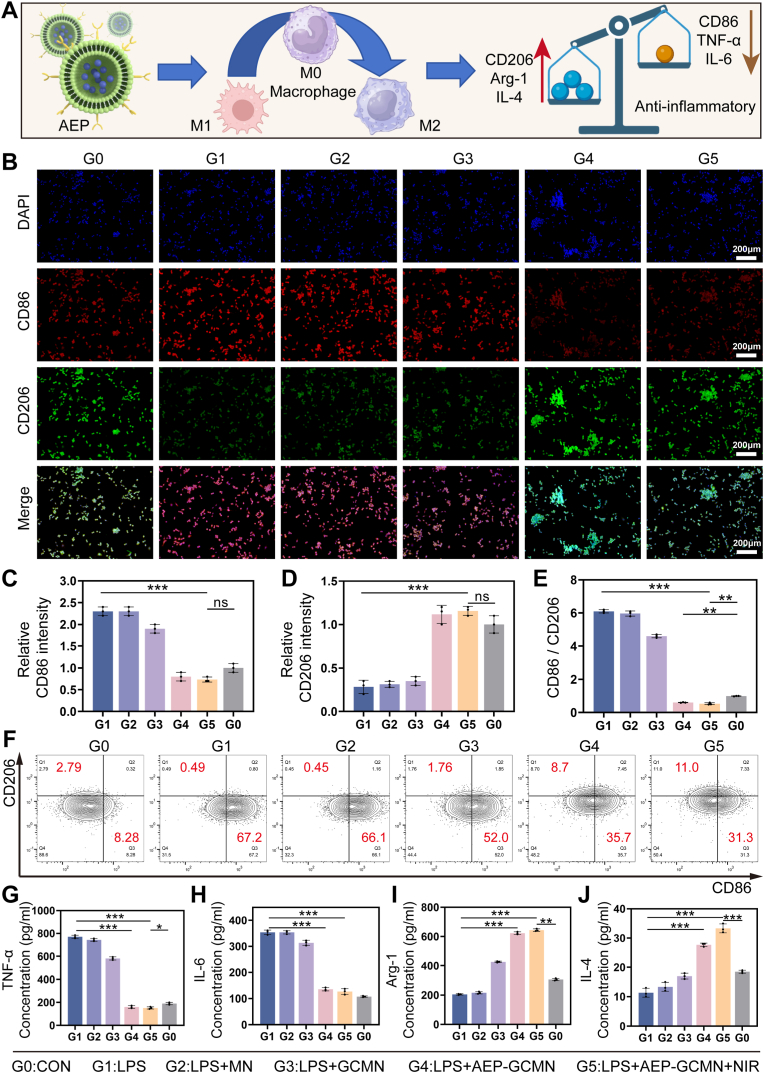
In vitro macrophage reprogramming by AEP-GCMN. (A) Schematic of AEP-induced M2 polarization. (B) Dual immunofluorescence staining of CD86 (red, M1) and CD206 (green, M2). Scale bar, 50 μm. (C-E) Quantitative analysis of CD86 intensity, CD206 intensity, and CD86/CD206 ratio. (F) Flow cytometry analysis of macrophage polarization. (G-J) ELISA quantification of TNF-α, IL-6, Arg-1, and IL-4 in culture supernatants. ∗*P* < 0.05, ∗∗*P* < 0.01 and ∗∗∗*P* < 0.001. (For interpretation of the references to color in this figure legend, the reader is referred to the Web version of this article.)

Chronic inflammation is a core pathological feature of non-healing DWs, characterized by persistent infiltration of pro-inflammatory M1 macrophages and insufficient transition to reparative M2 macrophages. To reverse this imbalance, the AEP-GCMN microneedle system was designed to actively regulate macrophage polarization. As illustrated in Fig. 5A, the engineered exosomes AEP, sustainably released from the core layer, can be internalized by macrophages. By delivering active components such as proanthocyanidins, AEP aims to promote macrophage transition towards the M2 phenotype, thereby upregulating repair-associated markers like CD206, Arg-1, and IL-4, while downregulating pro-inflammatory factors such as CD86, TNF-α, and IL-6.

To validate this regulatory effect, we established an inflammation model in macrophages (RAW264.7) using lipopolysaccharide (LPS) and systematically analyzed six experimental groups: G0 (blank control), G1 (LPS), G2 (LPS + blank MN), G3 (LPS + GCMN), G4 (LPS + AEP-GCMN), and G5 (LPS + GCMN + NIR). First, phenotypic observation was performed via dual immunofluorescence staining for CD86 (M1 marker) and CD206 (M2 marker) (Fig. 5B). Compared to the G0 group, cells in the G1 group exhibited intense CD86 (red) fluorescence and weak CD206 (green) fluorescence, confirming successful LPS-induced M1 polarization. Quantitative analysis (Fig. 5C–E) revealed that the CD86 relative fluorescence intensity in the G4 and G5 groups was significantly lower than in the G1 group, while the CD206 intensity was markedly higher, leading to a substantial decrease in the CD86/CD206 ratio. Notably, the G5 group (combined with NIR irradiation) demonstrated the most pronounced shift towards M2 polarization, suggesting that the photothermal effect from the shell layer may synergize with the biological activity of the core-layer AEP to collectively optimize the intracellular signaling environment. Meanwhile, flow cytometry analysis further validated the reprogramming effect of AEP-GCMN on macrophages. As depicted in Fig. 5F, upon LPS stimulation, the proportion of CD86^+^CD206^-^ Raw264.7 cells was the highest in Group G1 (67.2%), whereas Groups G2 and G3 exhibited a diminished effect. In contrast, the populations of CD86^+^CD206^-^ cells were significantly reduced in AEP-coated groups G4 (35.7%) and G5 (31.3%).

As shown in Fig. 5G–J, we measured the secretion of key cytokines in the cell supernatant via ELISA. The results showed that compared to the G1 group, the G4 and G5 groups significantly reduced the release of the pro-inflammatory factors TNF-α and IL-6. Concurrently, the secretion of anti-inflammatory factors Arg-1 and IL-4 was significantly upregulated in the G4 and G5 groups, whereas the expression levels of anti-inflammatory factors in Groups G2 and G3 did not exhibit an upward trend. This clear pattern of decrease and increase comprehensively confirms, from a secretomics perspective, that AEP-GCMN can reprogram macrophages from a pro-inflammatory state to a reparative state [45].

Collectively, the evidence spanning cellular phenotype and molecular secretion demonstrated that the system, primarily through the sustained release of AEP from its core, effectively redirected macrophage polarization under inflammatory conditions. The underlying mechanism is likely multifaceted, involving the modulation of key signaling pathways such as NF-κB and Nrf2 by the delivered proanthocyanidins to quell excessive inflammation and activate repair programs [46]. Moreover, the mild photothermal effect elicited by the shell layer not only failed to impair the immunomodulatory capacity of AEP-GCMN but also further potentiated this process. This active immunomodulatory function is thus a central mechanism by which AEP-GCMN interrupted the chronic inflammatory cycle in DWs, facilitating the critical transition from the inflammatory to the proliferative healing phase and providing a robust cellular rationale for its in vivo therapeutic efficacy.

### 2.5 In vitro antioxidant activity of the AEP-GCMN microneedle system

**Fig. 6:**
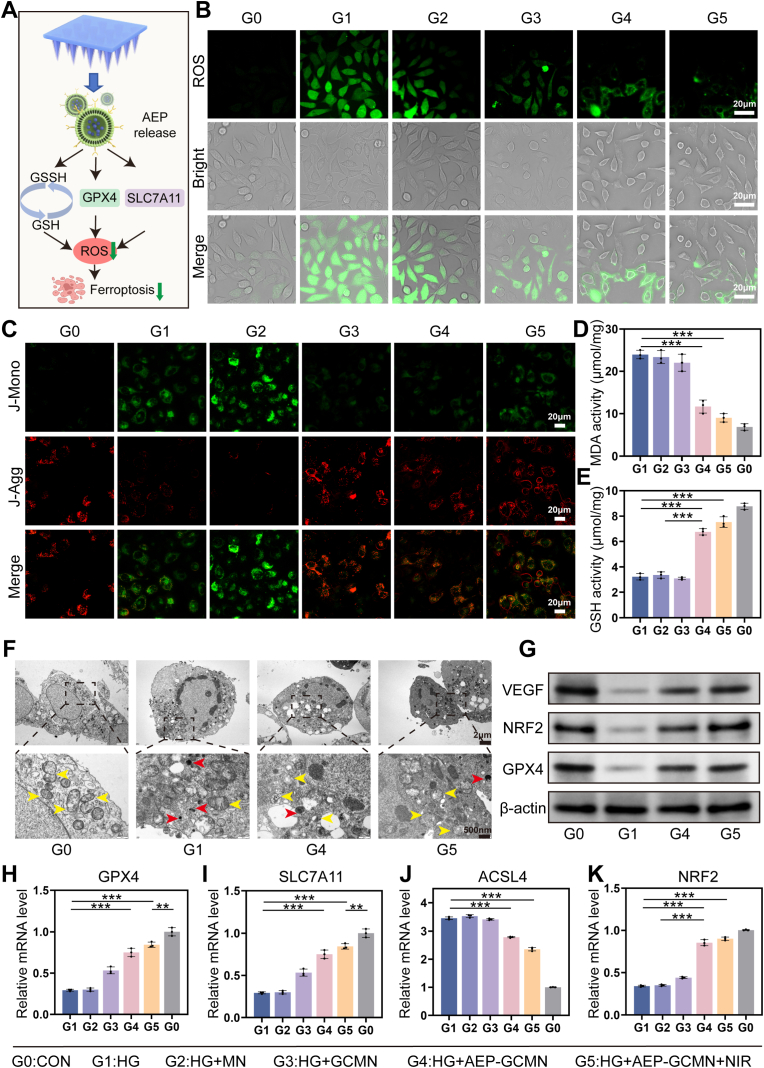
In vitro anti-ferroptosis activity of AEP-GCMN in high glucose (HG)-stimulated HUVECs. (A) Proposed mechanism of AEP-mediated inhibition of ferroptosis. (B) Immunofluorescence images of intracellular ROS. (C) JC-1 staining for mitochondrial membrane potential (red: aggregates, healthy; green: monomers, depolarized). (D, E) Quantification of MDA and GSH levels. (F) Representative SEM images of mitochondria. Arrows indicate ferroptotic morphology. (G) Western blot analysis of VEGF, NRF2, and GPX4. (H-K) qPCR analysis of GPX4, SLC7A11, ACSL4, and NRF2 mRNA expression. ∗*P* < 0.05, ∗∗*P* < 0.01 and ∗∗∗*P* < 0.001. (For interpretation of the references to color in this figure legend, the reader is referred to the Web version of this article.)

Ferroptosis, an iron-dependent form of regulated cell death driven by excessive lipid peroxidation, plays a critical role in endothelial cell injury within DWs [47]. The persistent high glucose environment in diabetic wounds serves as a primary catalyst for severe local oxidative stress through multiple overlapping metabolic pathways. Extensive literature indicates that the excessive influx of glucose into endothelial cells overloads mitochondrial metabolism leading to an accumulation of electron donors and the subsequent massive overproduction of superoxide radicals by the mitochondrial electron transport chain [48]. This profound mitochondrial dysfunction is further exacerbated by the accelerated formation of advanced glycation end products and the aberrant activation of the polyol pathway which collectively deplete endogenous antioxidant reserves. These cascade reactions persistently generate a substantial amount of reactive oxygen species and ultimately create a pathological microenvironment highly susceptible to lipid peroxidation and ferroptotic cell death. This study aimed to investigate whether the AEP-GCMN system can suppress ferroptosis by modulating the canonical GPX4 pathway, thereby mitigating high glucose (HG)-induced oxidative stress. As illustrated in Fig. 6A, we hypothesized that AEP released from the microneedles could elevate intracellular glutathione (GSH) levels, upregulate the expression of glutathione peroxidase 4 (GPX4) and solute carrier family 7 member 11 (SLC7A11), thereby effectively scavenging lipid ROS to inhibit ferroptosis and protect mitochondrial function.

To test this hypothesis, human umbilical vein endothelial cells (HUVECs) were subjected to HG treatment to mimic the metabolic stress in DWs, and six experimental groups were systematically evaluated (G0: blank control; G1: HG; G2: HG + blank MN; G3: HG + GCMN; G4: HG + AEP-GCMN; G5: HG + GCMN + NIR). As illustrated in Fig. 6B, immunofluorescence detection of intracellular ROS levels showed a significant increase in ROS signal in the G1 compared to the G0 group, and the green fluorescence intensity in groups G2 and G3 remained relatively high. In contrast, ROS fluorescence intensity was markedly suppressed in the G4 and G5 groups, with the most pronounced effect observed in G5 (combined with NIR irradiation), indicating that AEP-GCMN effectively alleviates HG-induced oxidative stress. JC-1 staining was used to assess mitochondrial membrane potential (Fig. 6C). HG treatment caused a decrease in potential (increase in green monomer fluorescence), while a partial recovery of red fluorescent aggregates was observed in the G4 and G5 groups, suggesting protected mitochondrial function.

As shown in Fig. 6D and E, oxidative stress markers in HUVECs were further analyzed. The results demonstrated that malondialdehyde (MDA), the terminal product of lipid peroxidation, was significantly increased in Group G1, whereas its levels were lower in Groups G4 and G5. Furthermore, glutathione (GSH)—an antioxidant that protects cells against oxidative damage—was present at higher levels in Groups G4 and G5 compared to the other groups. SEM provided direct evidence of organelle morphology (Fig. 6F). HG treatment caused marked mitochondrial shrinkage and increased membrane density in endothelial cells, presenting typical morphological features of ferroptosis. Following treatment with AEP-GCMN (G4, G5), mitochondrial morphology more closely resembled the normal state, with alleviated swelling and shrinkage.

At the molecular mechanism level, Western blot results demonstrated that HG treatment downregulated the protein expression of vascular endothelial growth factor (VEGF), nuclear factor erythroid 2-related factor 2 (NRF2), and GPX4 (Fig. 6G). Intervention with AEP-GCMN (G4, G5) upregulated the expression of these three proteins to varying degrees, suggesting the system concurrently promotes the synthesis of proteins crucial for angiogenic signaling, antioxidant defense, and anti-ferroptosis. qPCR quantification further confirmed this regulatory effect at the gene expression level (Fig. 6H–K): the G4 and G5 groups significantly upregulated the mRNA expression of the key anti-ferroptosis genes GPX4 and SLC7A11, as well as the master antioxidant regulator NRF2, while downregulating the expression of ACSL4, a positive regulator gene that promotes lipid synthesis during ferroptosis. Notably, the G5 group showed a superior protective trend over the G4 group across multiple indicators, suggesting a synergistic effect between the NIR-triggered photothermal response and the bioactivity of AEP in inhibiting ferroptosis. To further validate the inhibition of ferroptosis, we investigated the intracellular accumulation of ferrous iron, which acts as a primary catalyst for lipid peroxidation during ferroptotic cell death. We utilized the FerroOrange fluorescent probe to track the dynamic changes of intracellular ferrous iron levels across different treatment conditions (Fig. S5). The high glucose environment triggered a remarkable accumulation of intracellular ferrous iron in endothelial cells, presenting a classic functional signature of ferroptosis. Following intervention with the AEP-GCMN patch, the intense orange fluorescence signal was drastically diminished. This striking contrast confirms that the sustained delivery of AEP successfully mitigates intracellular iron overload.

Overall, the AEP-GCMN patch could effectively inhibit HG-induced endothelial cell ferroptosis. The core mechanism lies in the activation of the NRF2 signaling pathway by AEP released from the core layer, which upregulates downstream GPX4 and SLC7A11 to reduce lipid peroxidation and preserve mitochondrial structure and function, which is associated with PC coated in AEP activating the Nrf2 pathway to upregulate GSH levels in cells, thereby enhancing their antioxidant capacity [49]. The photothermal effect from the shell layer may further optimize this cytoprotective process through mild thermal stimulation.

### 2.6 Transcriptomic profiling unveils a coordinated regulatory network

**Fig. 7:**
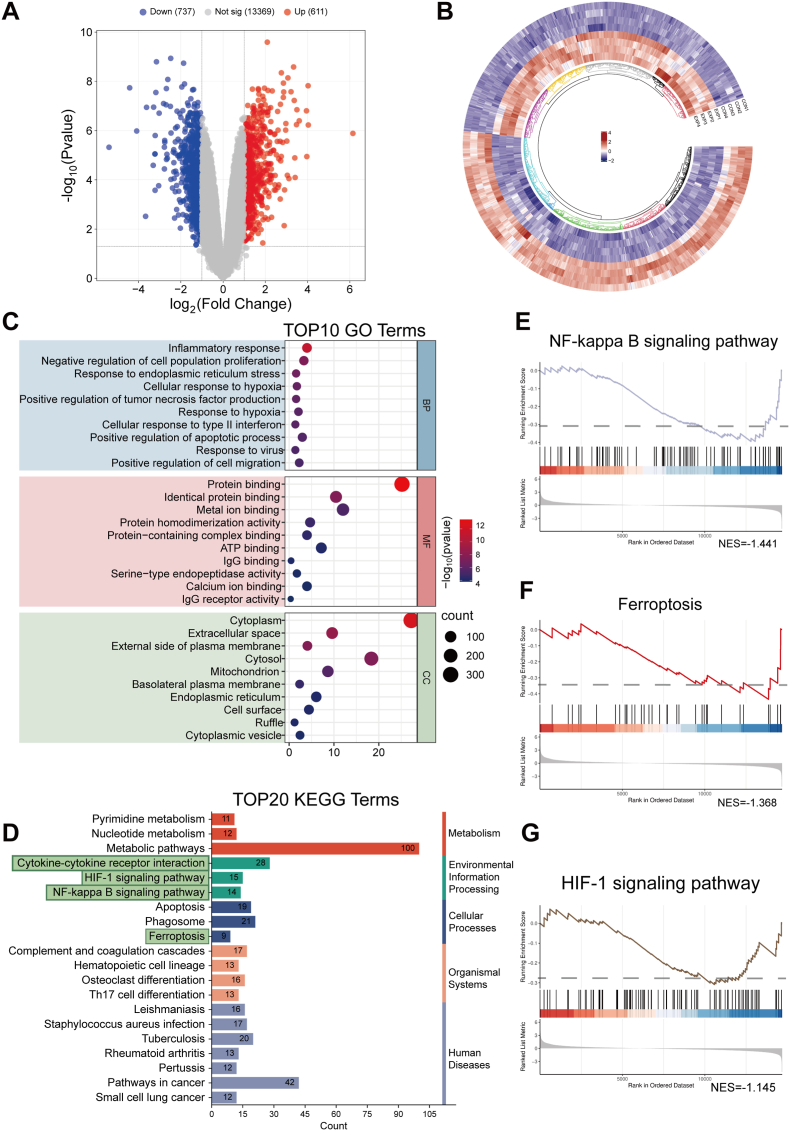
Transcriptome reprogramming and functional enrichment induced by AEP-GCMN + NIR treatment. (A) Volcano plot of differentially expressed genes (DEGs). (B) Circular heatmap of DEGs. (C) Bubble chart of Gene Ontology (GO) enrichment analysis. The top ten significantly enriched GO terms in the categories of Biological Process, Molecular Function, and Cellular Component are shown. (D) Top 20 significantly enriched KEGG pathways of DEGs. (E, F, G) Gene Set Enrichment Analysis (GSEA) results for the “NF-κB signaling pathway”, “Ferroptosis”, and “HIF-1 signaling pathway”, respectively, showing the global enrichment trends and scores of the relevant gene sets.

Following treatment with AEP-GCMN + NIR, a total of 1,348 differentially expressed genes (DEGs) were identified compared to the control group, with 611 significantly upregulated and 737 significantly downregulated, indicating that AEP-GCMN + NIR substantially reshaped the cellular transcriptional landscape (Fig. 7A). Circular heatmap analysis revealed a modular clustering pattern among these DEGs, suggesting functional co-regulation (Fig. 7B).

Gene Ontology (GO) enrichment analysis demonstrated that the DEGs were primarily involved in key stress and inflammation-related processes. Within the biological process category, they were significantly enriched in terms such as “inflammatory response,” “response to endoplasmic reticulum stress,” “response to hypoxia,” and “tumor necrosis factor production” (Fig. 7C). These results strongly imply that AEP-GCMN + NIR treatment inhibits endoplasmic reticulum stress and oxidative stress while suppressing canonical inflammatory pathways. At the cellular component level, DEGs were enriched in the “endoplasmic reticulum” and “extracellular region,” further corroborating the modulation of stress responses and cytokine secretion [50]. Collectively, these findings support the potential anti-inflammatory and oxidative stress-regulating roles of AEP-GCMN.

Kyoto Encyclopedia of Genes and Genomes (KEGG) pathway enrichment analysis revealed that the DEGs were most prominently enriched in the HIF-1 signaling pathway, ferroptosis, cytokine-cytokine receptor interaction, and Th17 cell differentiation pathways (Fig. 7D). Notably, the significant enrichment of the ferroptosis pathway provides key evidence for the direct targeting of this process by AEP-GCMN + NIR.

Gene Set Enrichment Analysis (GSEA) further elucidated the coordinated activation of these core pathways (Fig. 7E, F, G). Gene sets associated with the NF-κB signaling pathway and ferroptosis pathway showed significant and coordinated enrichment trends in the treatment group, with normalized enrichment scores (NES) of 1.442 and 1.368, respectively. Concurrently, alterations in Th17-related pathways may also be linked to the regulation of macrophage functional polarization.

In summary, the transcriptomic analyses suggest that AEP-GCMN + NIR may exert its coordinated anti-inflammatory and oxidative stress-relieving effects by targeting and modulating the ferroptosis process, thereby influencing key signaling axes such as HIF-1 and NF-κB.

### 2.7 In vivo evaluation of DW healing with the AEP-GCMN microneedle system

**Fig. 8:**
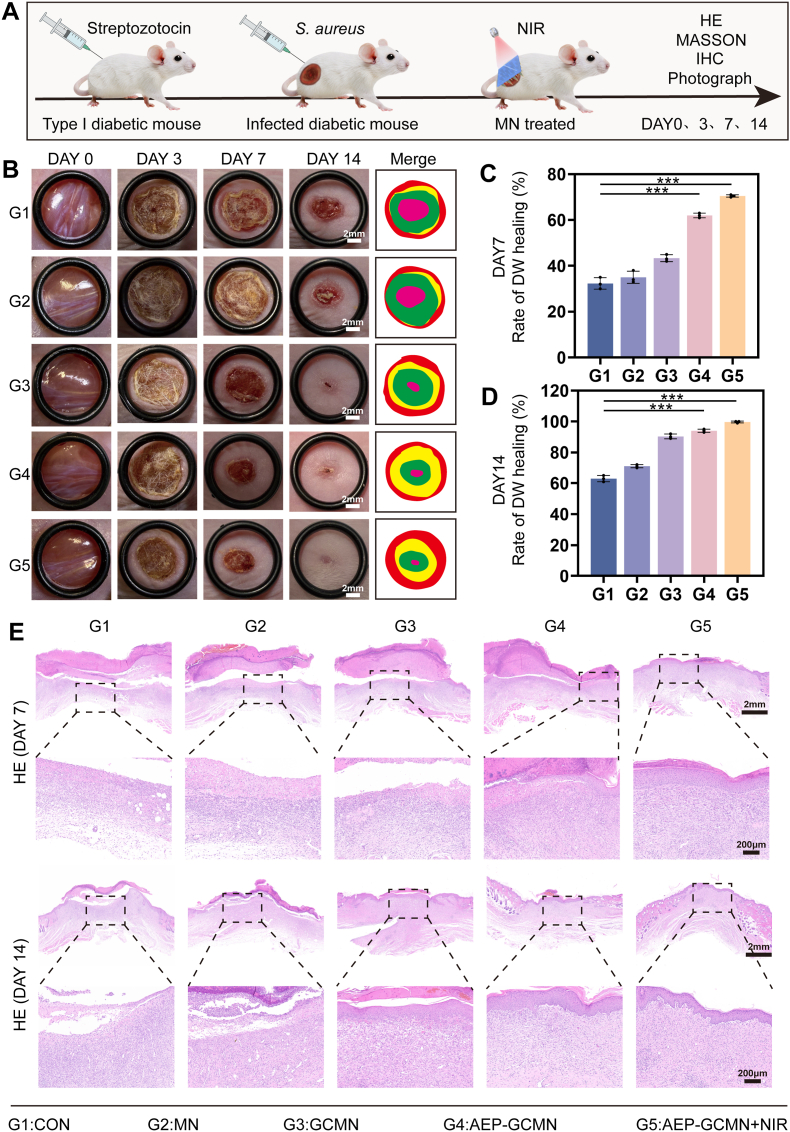
In vivo DW healing after treatment. (A) Schematic of the experimental procedure: STZ-induced diabetic mice with full-thickness wounds inoculated with *S. aureus* were treated and monitored. (B) Representative photographs of wounds from different treatment groups at various time points. (C, D) Quantitative analysis of wound healing rate on day 7 (C) and day 14 (D). (E) Representative H&E staining of wound sections on days 7 and 14. ∗*P* < 0.05, ∗∗*P* < 0.01 and ∗∗∗*P* < 0.001.

Following comprehensive in vitro validation of biosafety and functionality, this study established a complex in vivo pathological model to directly assess the dynamic wound-healing efficacy of the AEP-GCMN patch under conditions simulating clinical DWs. As shown in Fig. 8A, a type 1 diabetic mouse model was first established using streptozotocin (STZ) induction. Subsequently, full-thickness skin defect wounds were created on the dorsum and inoculated with *S. aureus* to mimic the core pathological features of diabetic infected wounds (hyperglycemia, ischemia, infection, and chronic inflammation). Animals were randomly divided into six treatment groups: G1 (blank control), G2 (blank MN), G3 (GCMN), G4 (AEP-GCMN), and G5 (AEP-GCMN + NIR irradiation). Prior to evaluating the wound healing efficacy, the successful establishment of the diabetic model was confirmed by systematically monitoring the blood glucose levels of the STZ induced mice. The monitoring results detailed in Fig. S6 of the Supporting Information reveal that the blood glucose concentrations of the selected animals consistently exceeded 16.7 mmol/L throughout the pre operative observation period. This sustained hyperglycemia validates that the experimental mice were in a reliable diabetic state.

As shown in Fig. 8B, macroscopic monitoring of the wound healing process clearly revealed dynamic differences among the groups, while the wound healing rates in the various groups were quantitatively analyzed at 7 and 14 days post-operation. By postoperative day 7, the wound healing rates of Groups G1, G2, and G3 were 32.28%, 35.69%, and 43.07%, respectively, all lower than that of Group G4 (60.94%). In contrast, the wound healing rate of Group G5 (69.98%) was significantly superior to those of the other groups. On day 14, Group G4 exhibited a favorable healing trend, suggesting the potential pro-repair activity of the engineered exosome AEP. Notably, the wounds in Group G5 were nearly fully closed, with a healing rate of 97.11% that was significantly higher than those of the other groups (Fig. 8C and D). This finding provides robust in vivo evidence for the synergistic interaction between the NIR-triggered photothermal effect of the shell and the biological activity of the core, which is highly consistent with the results of our in vitro antibacterial and cellular studies.

To deeply assess the quality of tissue regeneration, hematoxylin and eosin (H&E) staining was performed on wound tissues harvested on days 7 and 14. On day 7, wounds in the G1 and G2 groups still exhibited substantial inflammatory cell infiltration, marked tissue edema, and delayed re-epithelialization. The G3 group showed reduced inflammation, attributable to the continuous oxygen supply by CAT. In contrast, the G4 and G5 groups demonstrated a more favorable repair trajectory, with richer granulation tissue growth and more complete neo-epithelium. Crucially, the G5 group displayed the mildest inflammatory infiltration and the most rapid re-epithelialization (Fig. 8E), suggesting that the temporal synergy of photothermal antibacterial and AEP-mediated repair regulation effectively broke the infection-inflammation vicious cycle, creating a favorable microenvironment for tissue regeneration.

By day 14, these histological advantages became even more pronounced. Wounds in the G5 group had formed a continuous, complete stratified epithelial structure, and underpinned by mature granulation tissue, resembling normal skin architecture. In comparison, the other groups still exhibited issues such as varying degrees of residual inflammation or discontinuous epithelium.

The in vivo animal experiments thus demonstrate, at both macroscopic healing dynamics and microscopic tissue regeneration levels, that the AEP-GCMN microneedle system significantly accelerates the healing process of diabetic infected wounds and improves healing quality. The core therapeutic efficacy stems from the temporal synergy between the shell (GNS/CAT) and the core (AEP): the shell rapidly controls infection via photothermal effects and alleviates local hypoxia through enzymatic reaction, which is of great significance in the repair of DWs [51]. Subsequently, the sustained release of AEP from the core deeply modulates the wound microenvironment, driving orderly tissue regeneration through its multifaceted functions including anti-inflammatory, antioxidant, and pro-angiogenic activities.

### 2.8 Histological analysis of tissue repair and angiogenesis in vivo

**Fig. 9:**
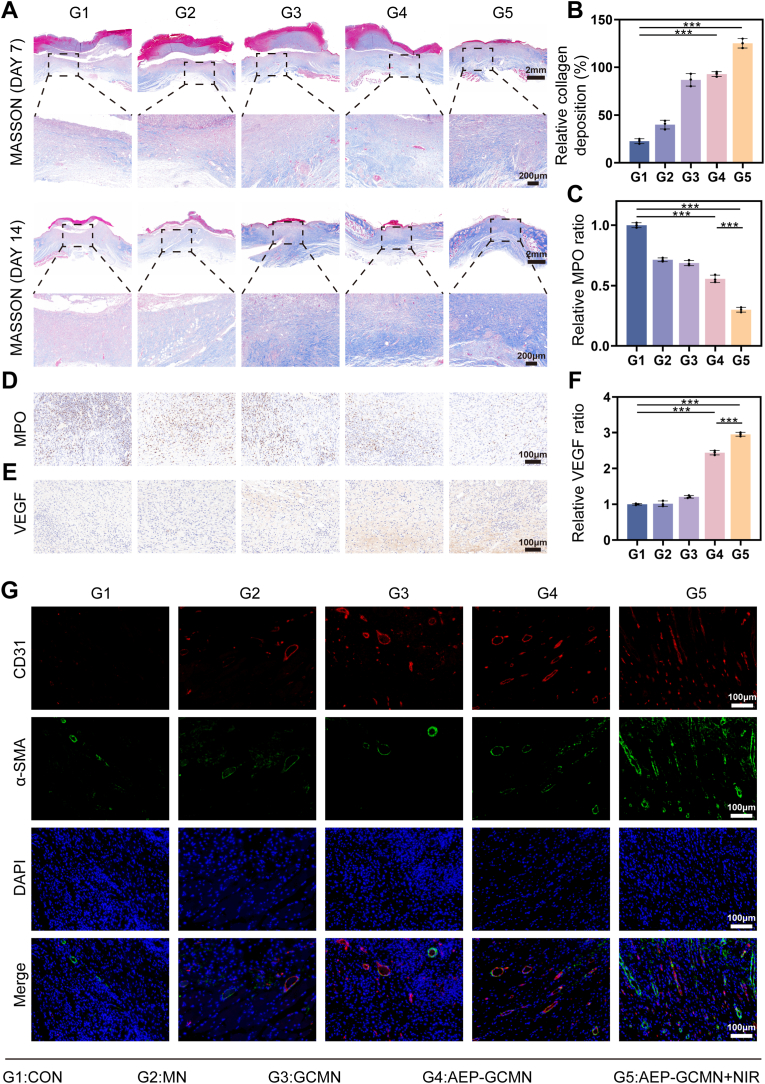
Histological evaluation of tissue repair and angiogenesis in DWs. (A) Representative Masson's trichrome staining of wound sections. (B) Quantification of collagen deposition area. (C) Quantitative analysis of myeloperoxidase (MPO) activity. (D) Representative MPO immunohistochemical staining. (E) Representative VEGF immunohistochemical staining. (F) Quantification of VEGF-positive area. (G) Dual immunofluorescence staining for CD31 (red, endothelial cells) and α-SMA (green, pericytes/smooth muscle cells), with merge showing co-localization. ∗*P* < 0.05, ∗∗*P* < 0.01 and ∗∗∗*P* < 0.001. (For interpretation of the references to color in this figure legend, the reader is referred to the Web version of this article.)

The results of Masson trichrome staining visually demonstrated the deposition and arrangement of collagen fibers among different groups, which serve as key indicators for assessing tissue regeneration and maturity [52]. As illustrated in Fig. 9A and B, compared with the control group, all treatment groups exhibited varying degrees of enhancement in collagen deposition on days 7 and 14. Specifically, the AEP-unloaded groups of G2 and G3 showed negligible changes relative to group G1 on day 7. In contrast, the collagen deposition rates of groups G4 and G5 were 2.16- and 3.09-fold that of group G1, respectively, indicating a substantial improvement. By day 14, collagen deposition in groups G2 and G3 had also improved significantly compared to the earlier time point, with an even more pronounced enhancement observed in group G4. Notably, group G5 demonstrated the most striking advantage, with a collagen deposition rate 4.90-fold that of group G1.

Immunohistochemical staining was performed to assess the inflammatory response and angiogenesis in the wound beds across different treatment groups. Myeloperoxidase (MPO) is a marker for neutrophil activity, and its level directly reflects the intensity of local tissue inflammation [53]. As shown in Fig. 9D, on day 7, the MPO level in group G1 was the highest, while no significant reduction was observed in groups G2 and G3. In contrast, the MPO level in group G4 was significantly lower than those in the aforementioned groups, whereas inflammation in Group G5 had essentially resolved (Fig. 9C). This result confirmed that the AEP-GCMN combined with NIR treatment can efficiently and rapidly clear neutrophil infiltration from the wound, creating a favorable low-inflammatory microenvironment for tissue regeneration. Regarding vascular regeneration, VEGF serves as the core signaling molecule mediating angiogenesis, and its expression level is critical for oxygen and nutrient delivery to regenerating tissues [54]. Immunohistochemical staining and corresponding quantitative analyses revealed that VEGF expression was significantly upregulated in Groups G4 and G5, with the most pronounced upregulation observed in Group G5 (Fig. 9E and F). This suggested that the sustained release of AEP from the core layer successfully activated the pro-angiogenic program in vivo, corroborating the in vitro findings.

To assess the quality of the newly formed vascular network, we conducted double immunofluorescence staining for CD31 and α-SMA on wound tissues harvested at day 7. CD31-positive signals reflect the density of nascent vessels, while co-localization with α-SMA indicates vascular maturity and stability. The results showed that the G5 group developed the densest and most structurally intact nascent microvascular network within the wound bed. These new vessels were not only abundant in number but also extensively surrounded by α-SMA-positive cells (Fig. 9G), indicating good vascular wall structure and functional maturity, which is more conducive to maintaining long-term blood supply [55].

In conclusion, the AEP-GCMN promotes high-quality wound repair through a multi-target synergistic mechanism: it simultaneously accelerates the deposition of a functional extracellular matrix, efficiently resolves excessive inflammation, and potently facilitates the reconstruction of a mature vascular network, and this therapeutic effect is amplified by the photothermal effect induced by NIR irradiation. This coordinated optimization of key tissue repair processes ultimately translates into the macroscopically observed rapid wound closure and the microscopically evident robust tissue regeneration.

### 2.9 Sustained immunomodulation and pharmacokinetic advantages of AEP-GCMN in vivo

**Fig. 10:**
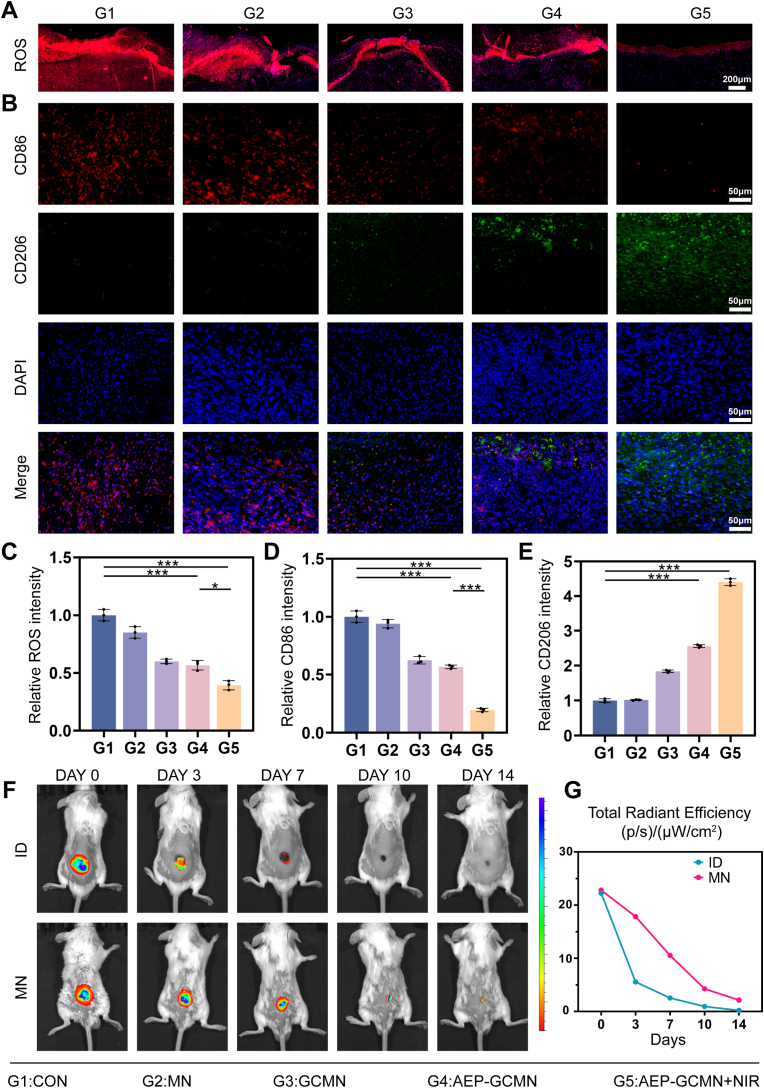
In vivo immunomodulation and pharmacokinetics of AEP-GCMN. (A) Representative fluorescence images of ROS in day-7 wound sections. (B) Dual immunofluorescence staining for CD86 (red, M1) and CD206 (green, M2) in day-7 wounds. (C-E) Quantitative analysis of ROS intensity (C), CD86^+^ area (D), and CD206^+^ area (E). (F) In vivo fluorescence images at various time points post-administration of DiR-labeled exosomes via intradermal injection (ID) or MN patch. (G) Quantitative fluorescence intensity curve from (F), showing sustained release kinetics of the MN group. ∗*P* < 0.05, ∗∗*P* < 0.01 and ∗∗∗*P* < 0.001. (For interpretation of the references to color in this figure legend, the reader is referred to the Web version of this article.)

As illustrated in Fluorescent Fig. 10A and C, staining for ROS in wound tissues revealed that on day 7, wounds treated with AEP-GCMN + NIR irradiation (Group G5) exhibited significantly lower ROS levels compared to other groups. This finding aligned with the in vitro observations that AEP could inhibit ferroptosis and upregulate antioxidant proteins, confirming the system's efficacy in alleviating the characteristic oxidative stress of DWs even within the complex in vivo environment.

The mitigation of oxidative stress lays the groundwork for the positive regulation of immune cell function in vivo. Dual immunofluorescence analysis of CD86 (M1 marker) and CD206 (M2 marker) in day-7 wound tissues showed that no significant differences were observed in the intensities of green and red fluorescence between group G1 and G2. However, compared with the control group, the red signal indicative of CD86 was attenuated, whereas the green signal indicative of CD206 was enhanced in groups G3 and G4. In contrast, group G5 exhibited the highest CD206^+^ signal and the lowest CD86^+^ signal (Fig. 10B, D and E). This directly demonstrated that the AEP-GCMN patch combined with NIR irradiation can effectively reprogram infiltrating macrophages from a pro-inflammatory M1 phenotype to a pro-reparative M2 phenotype during the critical inflammatory-proliferative phase of wound healing [56].

To visually compare the delivery efficiency of the AEP-GCMN patch with that of conventional administration approach, namely intradermal injection (ID), we labeled the engineered exosomes AEP with the near-infrared fluorescent dye DiR and administered them via either intradermal injection or the microneedle patch, followed by in vivo fluorescence imaging and tracking. As shown in Fig. 10F and G, the exosome signal in the ID group peaked rapidly after administration but subsequently decayed sharply, dropping to near-background levels by day 10. In contrast, the MN group exhibited a significantly prolonged sustained-release profile: its fluorescence signal remained at a stable plateau for a much longer duration, with a detectable signal still evident above background even on day 14. This result proved that the AEP-GCMN patch overcome the limitations of conventional methods and achieve sustained release of the therapeutic exosomes.

As shown in Supplementary Table S2, serum levels of alanine aminotransferase (ALT), aspartate aminotransferase (AST), and creatinine (Crea) in all treatment groups remained within the normal physiological range of healthy mice and showed no statistically significant increase compared with the control group. These combined results confirm that AEP-GCMN combined with NIR did not induce significant systemic organ toxicity.

Collectively, the above results indicated that the high in vivo efficacy of the AEP-GCMN system stems from its dual capability for rapid modulation and long-term maintenance. The synergy between its shell and core layers rapidly reduces wound oxidative stress and initiates macrophage polarization toward a reparative phenotype. Moreover, the unique architectural design of the MN ensures the prolonged retention and sustained release of the core therapeutic component, AEP, at the wound site, thereby extending the therapeutic window throughout the entire healing cascade. The combination of this spatiotemporally controlled drug delivery strategy and multi-mechanism synergistic effects offers an effective approach for modulating the microenvironment of DWs. To further benchmark the therapeutic performance of our AEP-GCMN system against existing MN-based strategies for DW healing, we compared our in vivo findings with three representative studies reported in recent years. Guo et al. developed a shark tooth-inspired MN patch for intelligent wound management, which achieved controlled drug release and motion monitoring but primarily focused on physical structural design without addressing the multi-stage pathological cascade of DWs [57]. Zhang et al. reported a dissolving and glucose-responsive insulin-releasing microneedle patch for type 1 diabetes therapy, which demonstrated glucose-responsive insulin delivery but targeted systemic metabolic control rather than local wound microenvironment modulation [58]. Zhao et al. prepared a photocatalytic and antibacterial MOF nanozyme (Au NCs@PCN) for infected DW healing, which exhibited excellent ROS generation and photothermal effects under NIR irradiation, achieving 95.3% and 90.6% killing rates against MRSA and AmprE. coli, respectively, and reducing wound coverage to 2.7% within 21 days in diabetic rats [59]; however, this system primarily focused on antibacterial action and lacked the spatiotemporal programmability and sustained exosome-mediated immunomodulation inherent to our system. our plant-derived aloe exosome-based approach offers advantages in scalability, low immunogenicity, and intrinsic pro-regenerative activity, while the core-shell design integrates photothermal therapy, enzyme therapy, and Exo-based therapy to achieve synergistic effects. This multi-mechanism combination improves DW management by enabling nearly complete wound closure, promoting the formation of mature vascular networks, and effectively alleviating chronic inflammation.

## 3 Conclusion

In this study, we successfully developed a core-shell structured MN patch (AEP-GCMN) that achieves spatiotemporally programmed, multi-stage intervention for DWs. The shell layer provides rapid oxygen generation and NIR-triggered photothermal antibacterial activity, while the core layer enables sustained release of engineered aloe-derived exosomes. Mechanistically, AEP activates the NRF2/GPX4 pathway to suppress endothelial ferroptosis, reprograms macrophages toward the M2 phenotype, and promotes angiogenesis and cell migration. In a murine model of infected diabetic wounds, the AEP-GCMN patch significantly accelerated healing, achieving nearly complete wound closure with enhanced collagen deposition, mature neovascularization, and effective resolution of chronic inflammation. The MN platform also ensured prolonged local retention of therapeutic exosomes. This rationally designed core-shell system enables intelligent, multi-stage intervention in the pathological cascade of diabetic wounds. Overall, the AEP-GCMN patch presents a promising and integrated strategy for the effective management of non-healing DWs and future work should focus on scaling up the production of plant-derived exosomes with consistent quality, evaluating the long-term biosafety and biodegradability of the patch, and ultimately translating this platform into clinical practice as a next-generation intelligent wound dressing.

## 4 Experimental section

### 4.1 Preparation and characterization of Aloe-Exo^PC^

Briefly, fresh *Aloe vera* gel was homogenized in cold PBS and sequentially centrifuged at increasing speeds, 300×*g* for 10 min, 2000×*g* for 20 min, and 10,000×*g* for 30 min at 4 °C to remove cellular debris and large vesicles. The final supernatant was ultracentrifuged at 120,000×*g* for 70 min at 4 °C to pellet the exosomes. The obtained pellet was resuspended in sterile PBS to obtain Aloe-Exos. Next, proanthocyanidins (MCE, 20347-71-1) were loaded into Aloe-Exos using an incubation method based on the permeability of the exosomal membrane. Briefly, purified PCs (0.25% w/v) were mixed with the Aloe-Exos suspension in PBS. The mixture was co-incubated at room temperature for 24 h under gentle agitation to facilitate passive diffusion and loading. Following incubation, the solution was centrifuged at 12,0000×*g* for 15 min. The resulting pellet was washed twice with PBS to obtain the purified Aloe-Exo^PC^. The engineered exosomes were then characterized for morphology and size distribution using nanoparticle tracking analysis (NTA) and transmission electron microscopy (TEM). To evaluate cellular internalization, the Aloe-Exos^PC^ were labeled by incubating with the lipophilic fluorescent dye Dil for 24 h, yielding Dil-AEP. HUVECs cells were then incubated with the Dil-AEP for 6 h. Subsequently, cells were fixed, permeabilized, and stained with DAPI and FITC-phalloidin to visualize nuclei and cytoskeleton, respectively. Finally, cellular uptake was observed and imaged using a confocal laser scanning microscope (CLSM).

### 4.2 Preparation of AEP-GCMN patch

Synthesis and Characterization of Gold Nanostars (GNS): Briefly, to prepare gold seed solution, 1.0 mL of chloroauric acid aqueous solution (10 mM) was rapidly added to 100 mL of cetyltrimethylammonium bromide (CTAB) aqueous solution (0.1 M) under vigorous stirring, followed by the addition of 0.6 mL of freshly prepared sodium borohydride solution (10 mM). The solution color changed rapidly from pale yellow to brownish-red. After further stirring for 5 min, the mixture was allowed to stand at room temperature for 2 h to obtain the gold seed solution. Subsequently, the growth solution was prepared by sequentially mixing 10 mL of CTAB (0.1 M), 1 mL of chloroauric acid (10 mM), 200 μL of silver nitrate (10 mM), and 100 μL of ascorbic acid (0.1 M) to homogeneity. Then, 1 mL of the gold seed solution (from the above preparation) was added to the growth solution. After gentle mixing, the reaction was allowed to proceed undisturbed for 3 h. Upon completion, the product was collected by centrifugation at 12,000 rpm for 15 min. The resulting precipitate was washed three times with deionized water to remove excess CTAB, yielding purified GNS colloid. The morphology of the synthesized GNS was examined by TEM. Finally, to prepare GNS at distinct target concentrations, the gold element concentration of the purified GNS stock solution was first accurately quantified via inductively coupled plasma mass spectrometry (ICP-MS). Subsequently, serial precise dilutions were performed using ultrapure water to facilitate subsequent photothermal experiments.

Fabrication of Core-Shell MN Patch: A polyvinyl alcohol (PVA) and polyvinylpyrrolidone (PVP) blend at an 8:2 mass ratio was co-dissolved in deionized water under stirring in a 90 °C water bath to prepare a homogeneous blend solution with a total polymer concentration of 30% (w/v). After cooling to room temperature, the prepared 20 μg/mL catalase (MACKLIN, 9001-05-2) was incorporated into the PVA/PVP solution to form the shell polymer solution. Separately, 100 mg of methacrylated hyaluronic acid (HAMA) was dissolved in 1 mL of sterile PBS to obtain a 10% (w/v) precursor solution. A photoinitiator was then added to this solution to a final concentration of 0.5% (w/v) and dissolved completely by stirring at 37 °C in the dark. Finally, the purified engineered exosome (AEP) suspension (30 μg/mL) was gently mixed and dispersed into the HAMA precursor solution to obtain the core prepolymer solution. The shell polymer solution was first dispensed into a polydimethylsiloxane (PDMS) MN mold. After centrifugation, the mold was then frozen at −20 °C to solidify the shell layer and subsequently dried under vacuum. Subsequently, the core-layer polymer solution was cast onto the pre-formed shell layer, followed by crosslinking and curing under ultraviolet (UV) light irradiation at a wavelength of 405 nm. After drying, the intact shell-core structured MN was immersed tip-down in the pre-prepared GNS dispersion at room temperature for 3 min. After retrieval, it was dried for 24 h, enabling the successful fabrication of AEP-GCMN patch, which was subsequently stored at 4 °C under dry and light-protected conditions.

### 4.3 Statistical analysis

Statistical analysis was performed with GraphPad Prism 9.0 software (GraphPad Software Inc., USA). All measurements were repeated three times by the same operator. Student's t-test was performed to determine statistical differences between two groups, and one-way ANOVA was used to calculate the differences across multiple groups. Data are shown as mean ± SD. ∗*P* < 0.05, ∗∗*P* < 0.01 and ∗∗∗*P*< 0.001, respectively. The * indicated the statistical difference between various groups.

## Funding

This work was supported by the Henan Medical Science and Technology Joint Building Program (20250014), and Peking union medical foundation-Ruiyi Emergency Medical Research Fund (PUMF01010010-2025–21).

## CRediT authorship contribution statement

**Xinyu Gu:** Data curation, Methodology, Writing – original draft, Writing – review & editing. **Shen Shen:** Formal analysis, Investigation, Visualization, Writing – review & editing. **Qingmiao Shi:** Conceptualization, Data curation, Methodology, Writing – original draft. **Ziyi Xu:** Investigation, Methodology, Software. **Minghang Zhang:** Conceptualization, Methodology. **Lifan Zhang:** Data curation, Software. **Chen Xue:** Methodology, Supervision. **Yuting He:** Data curation, Software. **Juan Lu:** Conceptualization, Methodology, Resources. **Li Li:** Funding acquisition, Project administration, Supervision, Writing – review & editing.

## Declaration of competing interest

The authors declare that they have no known competing financial interests or personal relationships that could have appeared to influence the work reported in this paper.

## Data Availability

Data will be made available on request.
