# Supplementary material for: Breaking the vicious cycle of diabetic wounds with an exosome-engineered dual-responsive microneedle patch
Source: Mater Today Bio. 2026 May 8;38:103198. doi: 10.1016/j.mtbio.2026.103198 (PMC13191656; doi:10.1016/j.mtbio.2026.103198)
Supplement: Multimedia component 1 [file mmc1.docx]

Supporting Information

Breaking the Vicious Cycle of Diabetic Wounds with an Exosome-Engineered Dual-Responsive Microneedle Patch

Xinyu Gu^A^, Shen Shen^B^, Qingmiao Shi^C^, Ziyi Xu^A^, Minghang Zhang^A^, Lifan Zhang^A^, Chen Xue^B^*, Yuting He^B^*, Juan Lu^D^*, Li Li^E^*

**Experimental Section**

**Characterization of AEP-GCMN patch**

The AEP-GCMN patch was sputter-coated with gold and observed using SEM, followed by elemental distribution analysis on the MN array via energy-dispersive X-ray spectroscopy (EDS). Fluorescently excitable MN patch was prepared by adding NHS-Cy5 and FITC to the shell and core precursor solutions, respectively, and the core-shell structure of the MN was further examined and imaged by CLSM.

**Mechanical Property Test of AEP-GCMN Patch**

To evaluate the mechanical properties of the MNs with different layer compositions, a texture analyzer was employed. Each MN (shell layer, core layer, and core-shell layer) was oriented with its tip upward and positioned on the horizontal platform of the testing instrument. A cylindrical probe was then vertically lowered at a predetermined speed, and the force applied to the MN was recorded to generate a force-displacement curve.

**In vitro transdermal test of AEP-GCMN patch**

The in vivo transdermal performance of the AEP-GCMN was evaluated using BALB/c mice. The patch was applied to the dorsal skin of the mice for 5 min, after which photographs of the skin were taken. The treated skin area was then excised from the mouse back, stained with DAPI, and observed under a fluorescence microscope to assess the penetration depth of the MN tips into the mouse skin.

**Photothermal Property of AEP-GCMN Patch**

MN patches containing varying concentrations of GNS were fabricated. Dried MN patch samples were secured on a thermal insulation sample stage. The initial temperature (T₀) was recorded using an infrared thermal imaging camera. Subsequently, the central area of each sample was irradiated perpendicularly with an 808 nm NIR laser at power densities of 0.5 and 1.0 W/cm² for a duration of 5 min. The temperature variation was dynamically monitored and recorded throughout the irradiation period. To assess the photothermal stability of the MN patches, the laser power density was fixed at 1.0 W/cm², and five complete "heating-cooling" cycles were performed. Before initiating the next heating cycle, the samples were allowed to cool for 5 min until they returned to the initial temperature. The entire process was continuously monitored using the infrared thermal imaging camera to document the temperature changes.

**In Vitro Release Experiment of Aloe-Exo^PC^**

To evaluate the release profile of AEP from the MN core layer, an in vitro release study was conducted. An MN patch (0.5 mg) containing AEP was immersed in 10 mL of PBS pre-warmed to 37°C and incubated under gentle shaking (100 rpm) to simulate physiological conditions. At predetermined time points (1, 2, 4, 6, 8, 10, 12, and 14 days), 1 mL of the release medium was withdrawn and immediately replaced with an equal volume of fresh, pre-warmed PBS to maintain sink conditions. The collected samples were centrifuged at 12,000 ×g for 15 min. The concentration of released AEP in the supernatant was determined by quantifying the total exosomal protein using a BCA protein assay kit (Thermo Scientific, USA). Absorbance was measured at 562 nm using a multimode microplate reader. The cumulative release percentage of AEP was calculated according to the following formula:

Cumulative Release (%) = (Mₜ / Mₜₒₜₐₗ) × 100%

**The antibacterial performance of AEP-GCMN**

Staphylococcus aureus (*S. aureus*) and Escherichia coli (*E. coli*) were cultured in a shaking incubator at 37 °C for 24 hours to reach the logarithmic growth phase. Subsequently, the bacterial suspensions were co-cultured with MN patches from different experimental groups, which had been pre-sterilized under UV light for 1 hour, for an additional 24 hours. In the near-infrared (NIR) group, the samples were exposed to an 808 nm NIR laser for 5 min every 8 hours to induce the photothermal effect. The bacterial suspensions from different groups were diluted, uniformly spread on agar plates, and incubated at 37 °C for 24 hours. The colony growth of both strains was documented photographically, and the bacterial survival rate was calculated based on the colony counts. To analyze the morphological changes in *S. aureus* and *E. coli*, SEM was employed. In detail, the treated bacterial samples were collected by centrifugation at 5,000 rpm, fixed with a 2.5% glutaraldehyde solution, and centrifuged to remove the supernatant. The pellets were then washed three times with PBS, dehydrated through a graded ethanol series, and finally dropped onto silicon wafers for SEM observation. Finally, using a SYTO9/PI Live/Dead Bacterial Staining Kit (FUSHENBIO, FS4005), the bacterial suspensions from different experimental groups were incubated in confocal dishes in the dark for 20 min according to the manufacturer's protocol. The samples were then examined and imaged using a CLSM.

The anti-biofilm efficacy of the samples was evaluated in vitro. Briefly, 1 mL of *S. aureus* or *E. coli* suspension at the logarithmic growth phase was inoculated into 6-well plates and incubated at 37 °C for 48 hours to allow biofilm formation. The pre-formed biofilms were then co-incubated with different sample groups for 24 hours. Then, the biofilms were gently washed twice with PBS and stained with 0.1% crystal violet solution for 20 min. The stained biofilms were photographed using a digital camera. For quantitative analysis, the bound dye was solubilized with 95% ethanol, and the absorbance was measured at 570 nm using a UV-Vis spectrophotometer to determine the anti-biofilm efficacy of each MN group. To investigate the three-dimensional architecture of the biofilms, biofilms of both bacterial strains were cultured in confocal dishes. After co-culture with different MN groups for 24 hours, the biofilms were gently rinsed twice with PBS and then stained using a SYTO9/PI Live/Dead Bacterial Staining Kit. The three-dimensional structure of the biofilms was then captured by CLSM.

**The in vitro biocompatibility of AEP-GCMN**

HUVECS cells were used to investigate the in vitro cytotoxicity of the AEP-GCMN patch. Specifically, different groups of MNs were immersed in DMEM containing 10% FBS to obtain MN-conditioned culture media. After HUVECS cells adhered to the culture plates, they were co-cultured with the different MN-conditioned media for 48 hours. Subsequently, the cells were stained using a Live/Dead Cell Staining Kit (Thermo, L3224) and observed by an inverted fluorescence microscope. In parallel, the viability of HUVECS cells co-cultured with the different MN-conditioned media was quantitatively assessed using a CCK-8 Assay Kit (Abcam, ab228554). To evaluate the hemocompatibility of the AEP-GCMN, MNs extracts were prepared by incubating the MN samples from different experimental groups in PBS. Then, 200 μL of freshly prepared mouse red blood cell suspension was added to the different MN extracts and incubated in a 37 °C water bath for 2 hours. Double-distilled water (ddH₂O) and PBS were used as the positive and negative controls, respectively. Following incubation, all samples were centrifuged at 1500 rpm for 5 min, and the supernatants were collected. The absorbance of each supernatant was measured at 500 nm using a multimode microplate reader, and the hemolysis rate was calculated as follows:

Hemolysis Rate (%) = (A_sample_ - A_negative_) / (A_positive_ - A_negative_) × 100%

**Scratch test**

A scratch wound healing assay was performed in 6-well plates to evaluate cell migration. After the HUVECS cells reached approximately 95% confluence following 48 hours of culture in DMEM, a uniform scratch was created in each well using a 200 μL pipette tip to form a clear linear gap. Subsequently, the cells were gently washed three times with PBS to remove detached cells and the cells were then incubated with different sample groups for 24 hours. Cell migration into the wound area was observed and monitored using a phase-contrast microscope. The images were analyzed using ImageJ software, and the percentage of wound closure over time was calculated.

**Transwell assay**

HUVECS cells at the logarithmic growth phase were seeded into the upper chambers of a 24-well Transwell plate at a density of 1 × 10⁴ cells per well. Then, 600 µL of DMEM or extracts from MNs of different groups were added to the corresponding lower chambers. The plate was incubated at 37°C with 5% CO₂ for 24 hours. After incubation, non-migrated cells on the upper surface of the membrane were gently removed using a cotton swab. The chambers were rinsed twice with PBS and fixed with 4% paraformaldehyde at room temperature for 20 min. Following another PBS wash, the cells were stained with 0.1% crystal violet solution for 20 min. Finally, excess dye was removed by washing with PBS. The migrated cells were observed, photographed by an inverted microscope, and quantified by cell counting using ImageJ software.

**Tube formation assay**

A tube formation assay was performed to evaluate the in vitro angiogenic activity. Briefly, pre-chilled Matrigel (Beyotime, C0372) was uniformly coated on the bottom of 24-well plates and incubated at 37 °C in a humidified chamber with 5% CO₂ for approximately 30 min to allow gel polymerization. Subsequently, serum-starved HUVECs were seeded onto the solidified matrix and co-cultured under different treatment conditions for 6 hours. Tube formation was observed and images were captured using an inverted microscope. The Number of network structures was quantified using ImageJ software.

**Immunofluorescence staining of Raw264.7 cells**

First, RAW 264.7 cells were seeded in 6-well plates at a density of 1 × 10⁵ cells per well. The cells were then stimulated with lipopolysaccharide (LPS) at a concentration of 100 ng/mL for 24 hours to induce M1 macrophage polarization. Subsequently, the stimulated RAW 264.7 cells were reseeded in confocal dishes at a density of 1 × 10⁵ cells per well and treated with samples from different experimental groups. Following treatment, the cells were fixed with 4% paraformaldehyde for 15 min at room temperature, permeabilized with 0.1% Triton X-100 for 10 min, and blocked with 1% bovine serum albumin (BSA) for 30 min at room temperature. The cells were then incubated overnight at 4°C with primary antibodies against CD86 (1:500 dilution, rabbit polyclonal antibody, ab239075, Abcam) and CD206 (1:1000 dilution, rabbit monoclonal antibody, 24595, Cell Signaling Technology). After washing with PBS, the cells were incubated with corresponding fluorescent secondary antibodies for 1 hour at room temperature in the dark. Finally, the cell nuclei were counterstained with DAPI, and the samples were mounted with an anti-fade mounting medium. Images were observed and captured using a laser scanning confocal microscope. The relative fluorescence intensity was quantified using ImageJ software.

**Flow Cytometry**

Cells from different treatment groups were collected and washed three times with PBS. Subsequently, the cells were co-incubated with CD206 antibody and CD86 antibody on ice for 30 min. Following incubation, the cells were washed twice with PBS. The fluorescence intensity of the target markers was measured using a flow cytometer and the acquired data were analyzed with FlowJo software.

**ELISA**

The supernatants of Raw264.7 macrophages treated differently were obtained by centrifugation and temporarily stored on ice. The concentrations of macrophage markers were detected using mouse-specific ELISA kits for TNF-α (Sigma-Aldrich, RAB0300), IL-6 (Beyotime, PI326), Arg-1 (Elabscience, E-EL-H0497) and IL-4 (Cusabio, CSB-E04634m) respectively. The absorbance of samples from different groups was measured, and then the concentrations of different samples were calculated through the standard curve.

**In Vitro ROS Scavenging Capacity of AEP-GCMN**

HUVECs were seeded in confocal dishes and cultured in a high-glucose environment using DMEM medium supplemented with 50 mM glucose. Following treatment with different groups of MNs, the cells were incubated with the DCFH-DA fluorescent probe in the dark for 20 min. After incubation, the cells were thoroughly washed with serum-free medium to remove residual probe, and fluorescent images were subsequently captured utilizing a CLSM.

**JC-1 staining**

The mitochondrial membrane potential (MMP) was assessed using the JC-1 MMP assay kit (Beyotime, C2006). Following various treatments, the culture medium was removed from HUVECs, and the cells were gently washed once with pre-warmed PBS. Then, 1 mL of freshly prepared JC-1 staining working solution was added to each well. The cells were incubated in the dark at 37°C in a 5% CO₂ incubator for 30 min. After incubation, the staining solution was discarded, and the cells were gently washed twice with pre-chilled JC-1 staining buffer (1×). Subsequently, 1 mL of complete culture medium was added to cover the cells. The cells were immediately observed under a CLSM, and the average fluorescence intensities of both red and green signals in each field of view were quantified using ImageJ software.

**MDA/GSH analysis**

Lipid peroxidation levels in HUVECs were assessed using MDA (Beyotime, S0131M) and GSH (Solarbio, BC1175) assay kits. Specifically, HUVECs from distinct treatment groups were harvested and lysed with pre-chilled cell lysis buffer. The lysates were then centrifuged at 4°C and 12,000 × g for 10 min to collect cell debris-free supernatants as test samples. Working solutions and standards were prepared in accordance with the kit instructions to generate standard curves. Finally, the absorbance values of the samples were measured and plotted against the standard curves to calculate the absolute concentrations of MDA and GSH in each sample.

**Analysis of the Ultrastructure of Mitochondria in HUVECs**

For observation of the ultrastructural morphology of mitochondria, HUVECs subjected to various treatments were sequentially processed through prefixation with glutaraldehyde, postfixation with osmium tetroxide, dehydration using a graded ethanol series, and infiltration/embedding in epoxy resin. Ultrathin sections were then prepared, double-stained with uranyl acetate and lead citrate to enhance contrast, and subsequently examined under a transmission electron microscope (TEM) for imaging.

**Western Blot analysis**

Total protein was extracted from treated cell or tissue samples using RIPA lysis buffer and quantified via the BCA method. Equal amounts of protein samples were separated by SDS-PAGE and subsequently transferred onto PVDF membranes. After blocking with 5% skim milk for 1 hour at room temperature, the membranes were incubated overnight at 4 °C with specific primary antibodies, including VEGF (1:1000, rabbit monoclonal antibody, D9W3P, Cell Signaling Technology), NRF2 (1:1000, rabbit monoclonal antibody, D9I4K, Cell Signaling Technology), GPX4 (1:5000, rabbit polyclonal antibody, ab125066, Abcam), and β-actin (1:1000, mouse monoclonal antibody, ab8226, Abcam). Following washing with TBST, the membranes were incubated with corresponding HRP-conjugated secondary antibodies, Goat Anti-Mouse IgG H&L (HRP) (1:2000, ab6789, Abcam), for 1 hour at room temperature. Finally, protein bands were visualized using an ECL chemiluminescence substrate, and band intensity was analyzed with Image Lab software. The relative expression levels of target proteins were calculated by normalizing to β-actin.

**Functional Annotation and Pathway Analysis**

Gene Ontology (GO) and Kyoto Encyclopedia of Genes and Genomes (KEGG) pathway enrichment were conducted with the clusterProfiler R package (version 4.10.0), where KEGG enrichment was specifically carried out using the enrichKEGG function. We applied the CIBERSORT algorithm coupled with a mouse-specific signature matrix to transcript-per-million (TPM) normalized expression data.

**Animal experimentation**

One hundred 6-8-week-old male BALB/c mice were purchased**.** All experimental procedures were conducted in accordance with the Guide for the Care and Use of Laboratory Animals (8th edition, 2011) and were approved by. The animals were housed under controlled conditions at 22 °C with 55–65% relative humidity, under a 12‑h light/12‑h dark cycle, with free access to food and water. This study was approved by the Institutional Review Board of The First Affiliated Hospital of Zhengzhou University (Approval No. 2025-KY-1996-001).

**Establishment of infected diabetic wound model**

Diabetes was induced in healthy BALB/c mice by intraperitoneal injection of streptozotocin (STZ). Mice with blood glucose levels persistently above 16.7 mmol/L for at least one month were defined as the diabetic model. These diabetic mice were then randomly divided into five groups (n = 10): Control group (wounds covered with sterile gauze only), MN group, GCMN group, AEP-GCMN group, and AEP-GCMN+NIR group. Prior to surgery, mice were anesthetized with isoflurane inhalation. A full-thickness circular skin wound with a diameter of 10 mm was created on the dorsal surface of each mouse using a biopsy punch. To establish an infected diabetic wound model, 1 mL of Staphylococcus aureus suspension (1 × 10^9^ CFU/mL) was injected into the wound bed. After receiving different treatments according to their group assignments, digital photographs of the wounds were taken on days 0, 3, 7, and 14. The wound area was measured using ImageJ software.

**Immunohistochemical staining and immunofluorescence staining**

For histological and immunological analysis, the injured dorsal skin areas from each group of mice were excised and cut into 10 mm × 12 mm strips. These tissue strips were fixed in 4% paraformaldehyde for 48 h, followed by dehydration and paraffin embedding. Consecutive sections of 5 μm thickness were prepared for Hematoxylin and Eosin (HE) staining and Masson's trichrome staining. For immunohistochemistry, sections were incubated overnight at 4°C with primary antibodies against MPO (1:100, ab9535, Abcam) and VEGF (1:500, 2463S, Cell Signaling Technology), followed by incubation with an HRP-conjugated goat anti-mouse secondary antibody (1:500, ab6789, Abcam) for 1 hour at room temperature. Images were observed under an optical microscope and quantitatively analyzed using ImageJ software. For immunofluorescence analysis, ROS levels in the wound tissue were assessed using a DHE ROS Detection Kit (Thermo, D11347). Additionally, sections were incubated overnight at 4°C with primary antibodies against CD86 (1:500, ab239075, Abcam), CD206 (1:500, 24595S, Cell Signaling Technology), CD31 (1:200, M0823, Agilent DAKO), and α-SMA (1:1000, A5228, Sigma-Aldrich). This was followed by incubation with the same HRP-conjugated secondary antibody (1:500, ab6789, Abcam) for 1 hour at room temperature. Finally, images were captured using a fluorescence microscope and analyzed with ImageJ software.

**IVIS**

To evaluate the sustained delivery and pharmacokinetic profile of AEP, purified AEP was labeled with the fluorescent dye DiR by co-incubation for 6 hours, resulting in Dil-labeled exosomes (Dil-AEP). Two delivery methods were compared: sustained release via GCMN and direct intradermal injection (ID) of AEP. Specifically, Dil-AEP was administered to the wound area of diabetic mice either by intradermal (ID) injection (ID group) or via a AEP-GCMN patch (MN group). Whole-body fluorescence images were acquired using an in vivo imaging system (IVIS) at days 0, 3, 7, 10 and 14 post-treatment. The fluorescence intensity within the wound region was subsequently quantified using Living Image software.


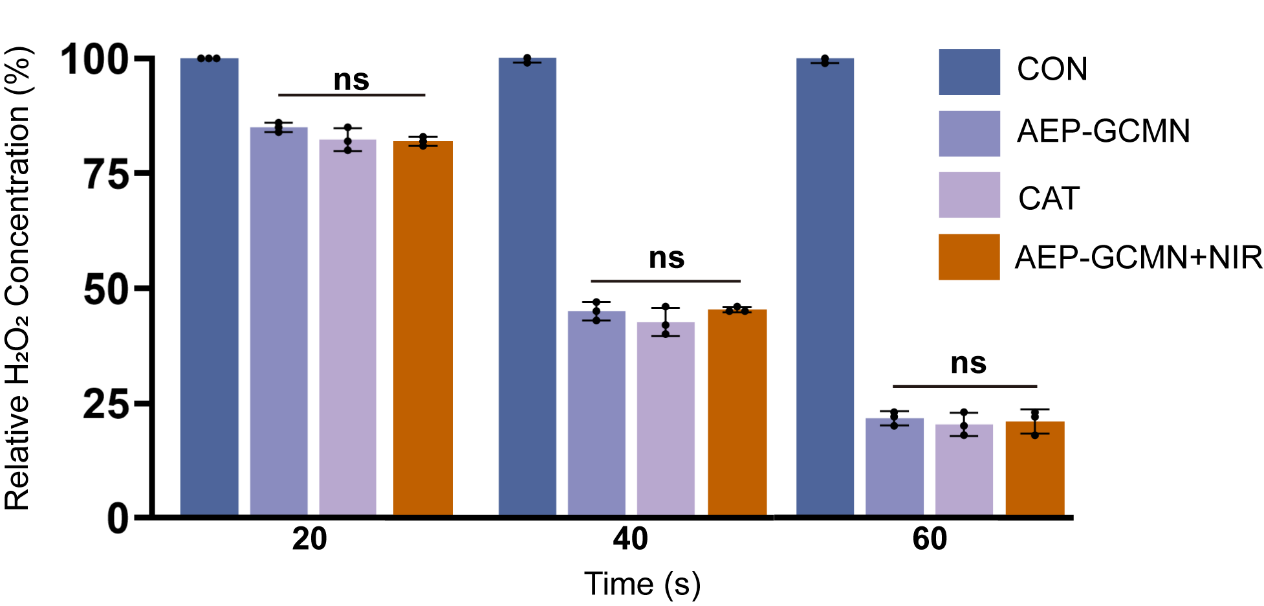


**Figure S1.** Catalytic activity validation of CAT released from the AEP-GCMN patch.


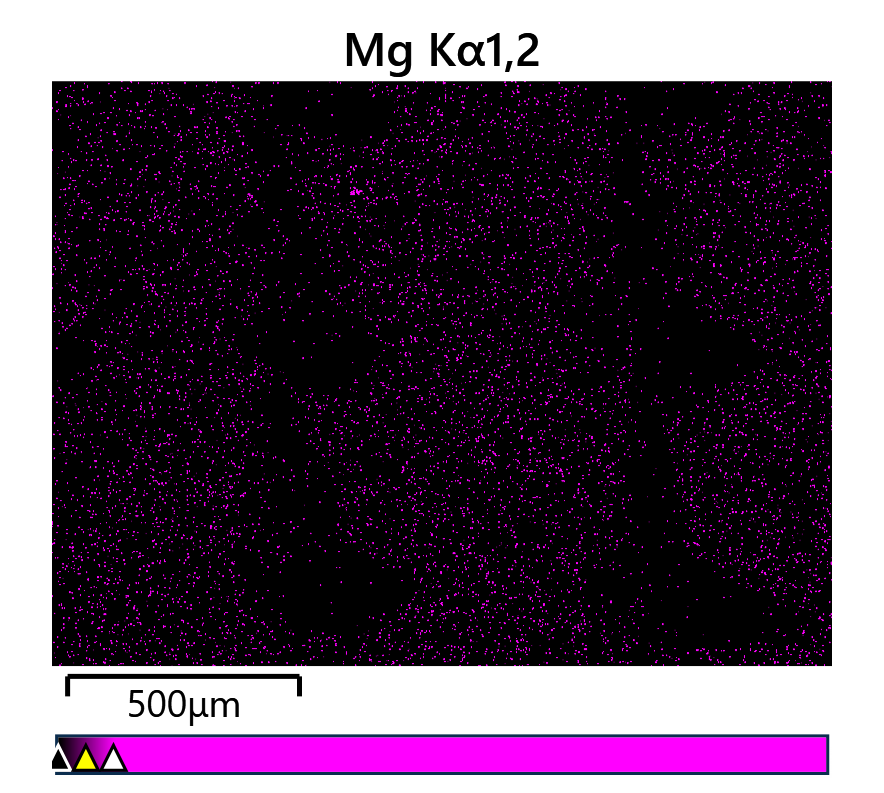


**Figure S2.** Surface elemental analysis(Au) of AEP-GCMN patch (Scale bar: 500 mm).


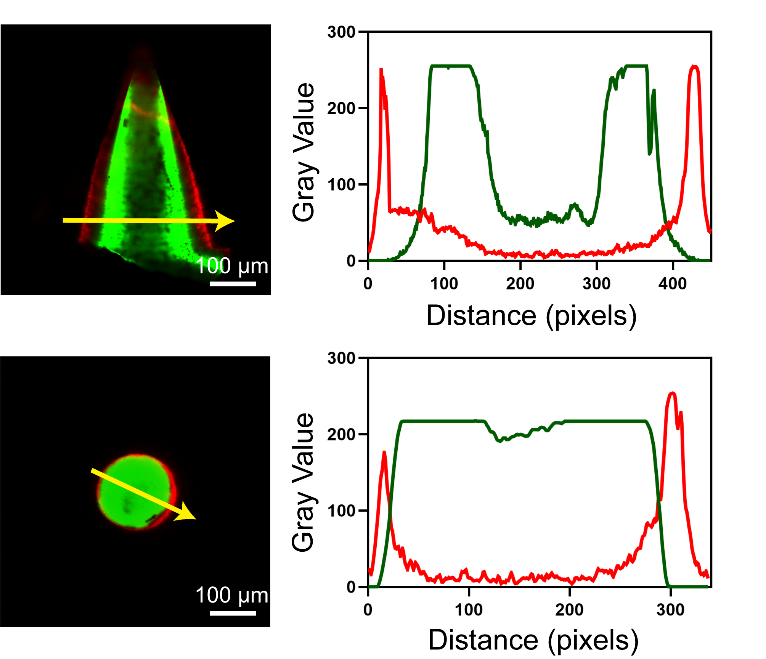


**Figure S3.** Colocalization analysis of AEP-GCMN patch fluorescence staining (Scale bar: 100 μm).

**
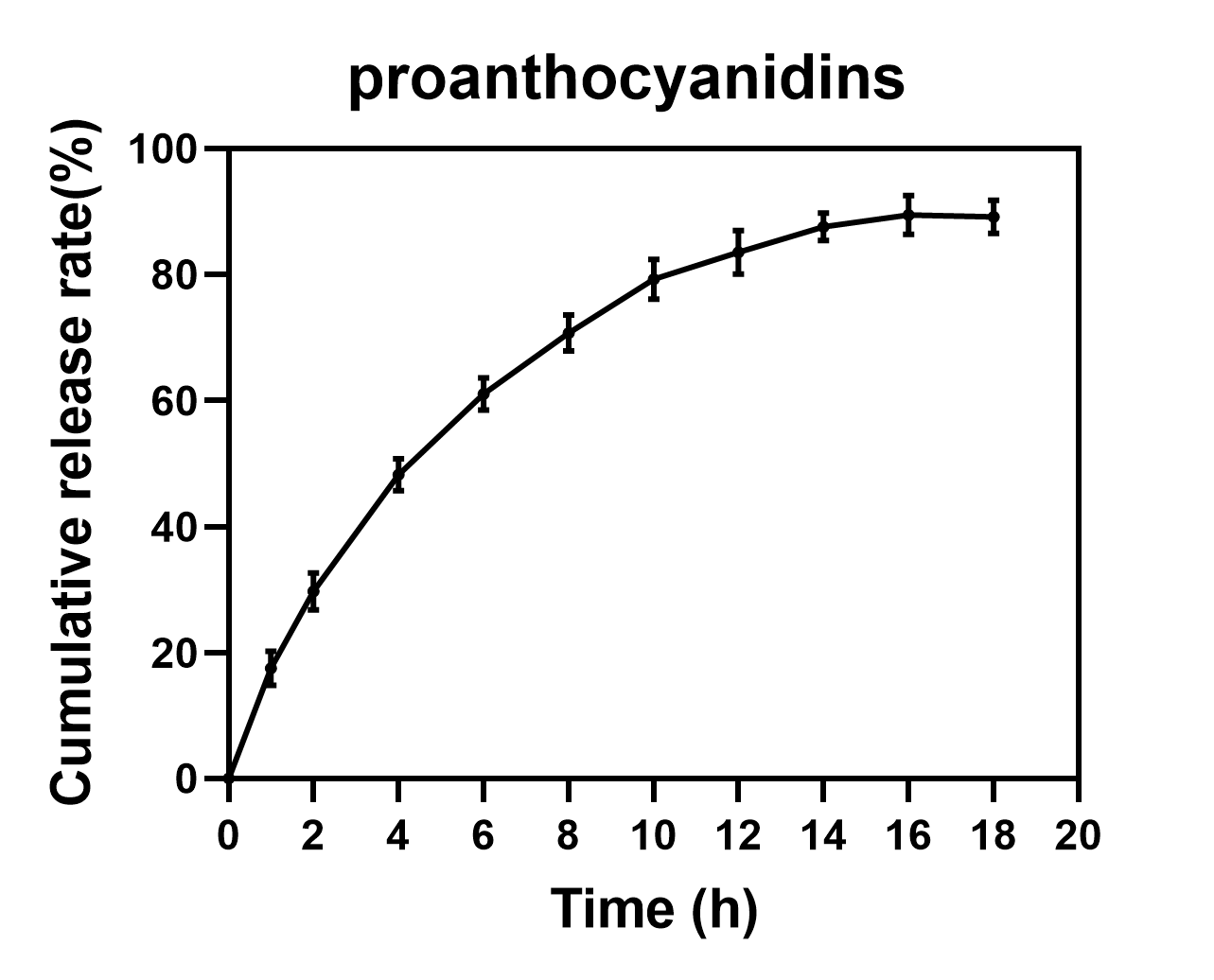
**

**Figure S4.** In vitro sustained release curve of PC from the AEP-GCMN microneedle patch in PBS at 37 °C. Data are expressed as mean ± SD (n = 3).

**
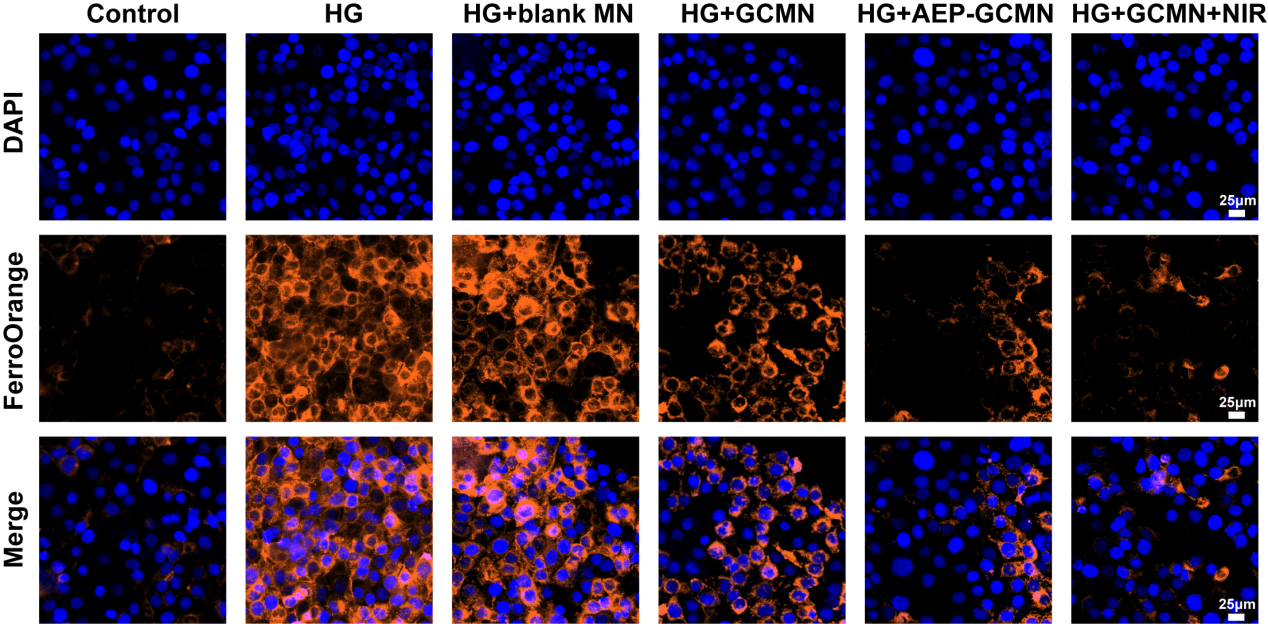
**

**Figure S5.** Representative FerroOrange staining images and corresponding quantitative analysis of intracellular ferrous iron (Fe^2+^) levels across various treatment groups. The dramatic reduction in orange fluorescence confirms the mitigation of high glucose-induced iron overload by the AEP-GCMN patch. Data are expressed as mean ± SD (n = 3). Scale bar = 25 μm.


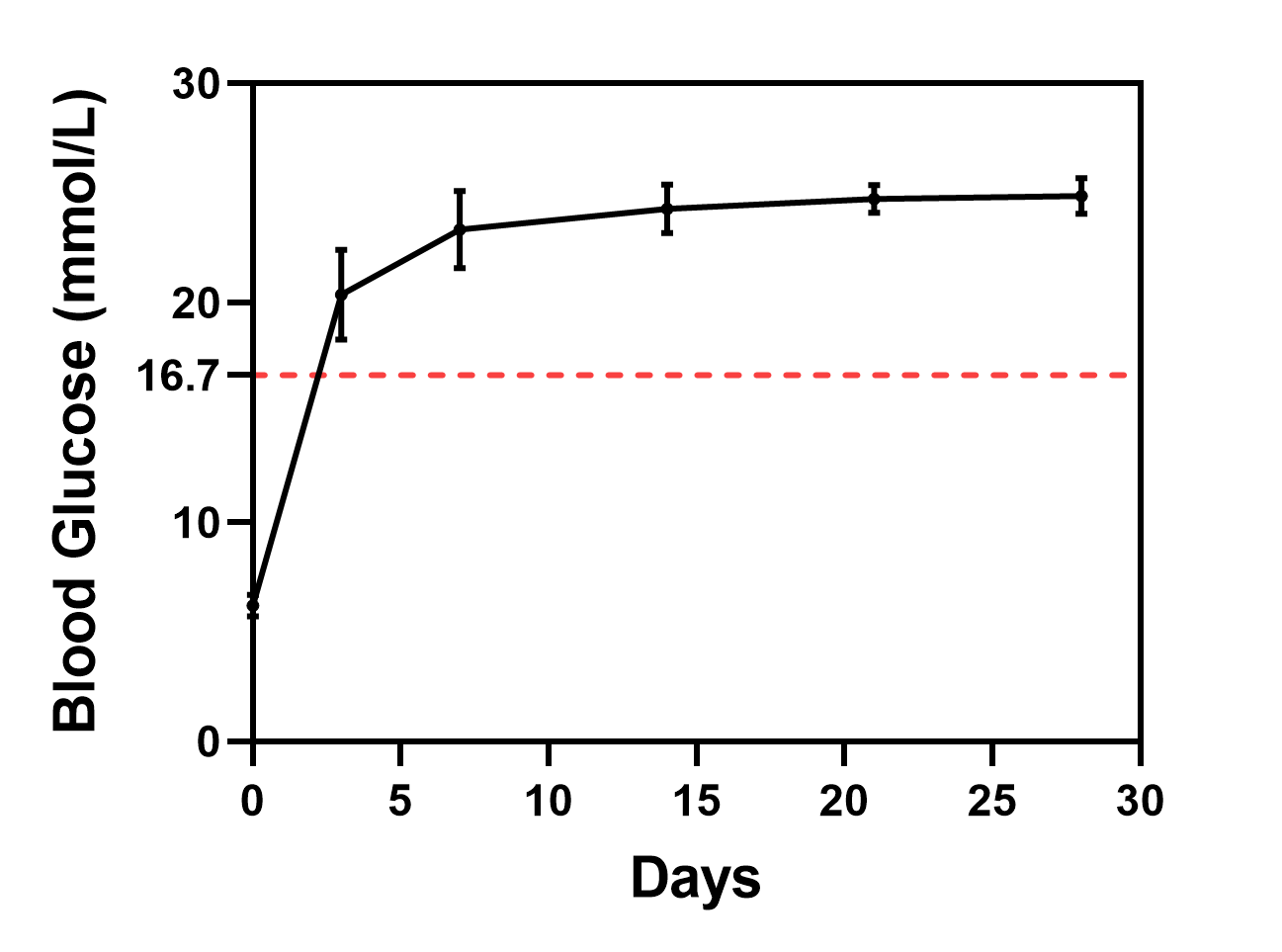


**Figure S6.** Validation of the STZ-induced diabetic mouse model. Dynamic monitoring of blood glucose levels in the STZ-treated group over a 28-day induction period. The red dashed line at 16.7 mmol/L indicates the diagnostic threshold for successful diabetic modeling.

**Supplementary Table 1.** (Quantification of proanthocyanidin loading into Aloe-Exo.)

| Sample | PC  Drug Loading Content (%) | PC  Encapsulation Efficiency (%) |
| --- | --- | --- |
| 1 | 15.21 | 18.53 |
| 2 | 14.95 | 19.52 |
| 3 | 13.83 | 21.41 |

**Supplementary Table 2.** (Serum biochemistry analysis.)

| Group | ALT (U/L) | AST (U/L) | ALP (U/L) | CREA  (μmol/L) |
| --- | --- | --- | --- | --- |
| CON | 42.2±5.6 | 160.5±14.8 | 268.1±24.5 | 15.5±3.7 |
| MN | 40.1±8.3 | 164.1±17.8 | 273.4±20.6 | 15.2±3.9 |
| GCMN | 45.2±6.5 | 162.8±16.5 | 275.1±20.5 | 15.2±3.3 |
| AEP-GCMN | 46.6±7.6 | 163.7±14.7 | 282.4±20.4 | 14.5±4.2 |
| AEP-GCMN+NIR | 48.5±7.3 | 175.5±15.8 | 254.3±25.5 | 16.2±6.3 |
